# Exploring Rates of Adherence and Barriers to Time-Restricted Eating

**DOI:** 10.3390/nu15102336

**Published:** 2023-05-16

**Authors:** Paul W. Jefcoate, M. Denise Robertson, Jane Ogden, Jonathan D. Johnston

**Affiliations:** 1 Section of Chronobiology, School of Biosciences, Faculty of Health and Medical Sciences, University of Surrey, Guildford GU2 7XH, Surrey, UK; j.johnston@surrey.ac.uk; 2 Nutritional Sciences, Faculty of Health and Medical Sciences, University of Surrey, Guildford GU2 7XH, Surrey, UK; m.robertson@surrey.ac.uk; 3 School of Psychology, Faculty of Health and Medical Sciences, University of Surrey, Guildford GU2 7XH, Surrey, UK; j.ogden@surrey.ac.uk

**Keywords:** time-restricted eating, TRE, chrononutrition, intermittent fasting, adherence, qualitative methods

## Abstract

Whilst the treatment and prevention of overweight and obesity-related disease is managed by restricting daily energy intake, long-term adherence to dietary strategies appears unsustainable. Time-restricted eating (TRE) aims to position energy intake in an eating window under 12 h per day and offers an alternative behavioral intervention, which can aid weight management and improve cardiometabolic health. Adherence to previous TRE protocols is estimated at between 63 and 100%, although the accuracy of reporting is unclear. This study therefore aimed to provide an objective, subjective, and qualitative overview of adherence to a prescribed TRE protocol, and to identify any potential barriers affecting adherence. Adherence after 5 weeks of TRE was estimated at ~63% based on continuous glucose monitoring data when compared with time-stamped diet diaries. Subjective participant responses reported adherence at an average of ~61% per week. Barriers to adopting TRE, including work schedules, social events, and family life, were identified by participants during qualitative interviews. The findings of this study suggest that the development of personalized TRE protocols may help to navigate the barriers to adherence leading to improved health-related outcomes.

## 1. Introduction

Treatment for obesity is broadly covered by dietary and physical interventions, cognitive therapy, pharmacological drugs, and bariatric surgery [1], although dietary interventions are administered mostly amongst individuals living with overweight or obesity [2,3]. Over the last few decades, public health strategies have focused primarily on adjusting the macronutrient content, quantity, and quality of the diet to promote continuous energy restriction (CER), leading to clinically significant weight loss and subsequent improvements to health. Although clinical trials report that sustained weight loss of >10–15 kg after 12 months leads to greater rates of diabetes remission, lower HbA_1C_, and decreased mean triglycerides [4], the availability of a dietary intervention promoting and maintaining weight loss under real-life conditions remains elusive.

Intermittent energy restriction (IER) has emerged as an alternative strategy to CER, focusing on daily food abstinence followed by concise periods of ad libitum eating. There are three main versions of IER, including alternate day fasting (ADF), the 5:2 diet, and time-restricted eating (TRE). The primary aim of TRE is to restrict all daily energy intake into a timeframe lasting no more than 12 h, with less emphasis on calorie counting or adjusting diet quantity or quality. This dietary strategy focusing on the timing of energy consumption has been highlighted as a potential treatment demonstrated to reduce body weight, body mass index (BMI), total body fat percentage, waist circumference, and hip circumference in individuals with overweight and obesity [5,6,7,8,9,10,11], as well as fat mass in healthy-weight, resistance-trained males and females [12,13,14].

There are numerous TRE strategies, which vary in the duration of their eating windows from ~4 to 12 h, distributed at different times throughout the day. What remains unclear are the mechanisms driving these health-promoting outcomes. TRE has been demonstrated to result in weight loss ranging from 1% to 4% of body weight in studies lasting 4 weeks [15], 8 weeks [7], 12 weeks [6,8,11] and 16 weeks [9], with a concomitant reduction in daily energy intake of between ~8% and ~20% in studies allowing ad libitum eating [8,9]. Additionally, TRE may modify substrate oxidation, utilizing free fatty acids and fatty-acid derived ketones as energy by extending the fasting period to ≥12 h, leading to subsequent reductions in body adiposity [5,16,17]. Other TRE programs suggest there are cardiometabolic benefits associated with restricted eating, specifically in improving postprandial or 24 h mean glucose levels, fasting glucose, and insulin levels when early TRE (eTRE) is followed [18,19,20,21,22].

Attrition rates, however, are a consistent issue associated with weight loss-related dietary interventions [23]. A review of popular dietary strategies designed to induce an energy deficit showed that they were equally effective at stimulating weight loss, suggesting that individual preferences should be considered to strengthen rates of adherence to nutrition interventions [24]. Much of the TRE literature to date has focused on measuring adherence to the respective protocols, with success rates ranging from 63 to 100% [5,6,7,8,12,13,14,15,17,18,19,20,21,25,26]. What remains unknown is the validity of these claims, since adherence is frequently measured using self-reported methods. Moreover, the application of clinical methods may not be compatible within the reality of modern-day living. Research indicates that adherence to a diet is greater in studies when foods are provided within a clinical setting or available to take home [3,4,5]. This may partially explain why compliance rates of ~98% were observed in a 5-week eTRE study wherein all meals were provided and consumed within a 6 h eating window between 9:00 and 15:00 h [21]. Patients with type 2 diabetes were adherent on ~6 d weekly over a 12-week period of eating ad libitum between 8:00 and 18:00 h [27], and similar compliance rates occurred under 4 h and 6 h TRE conditions in adults with obesity [7]. Subjects adhering to an 8 h eating window within ±15 min, ±30 min, and ±60 min were able to do so for 55.5%, 60% and 66.3% of the time, respectively [6]. However, a 14-week study in physically active males and females hypothesized that adherence would need to be more than 70% to acquire the benefits of TRE. In that study, participants were compliant with the TRE regime on 5 days/week (71%), in comparison to 4.5 days/week (64%) for the controls [28]. In response to the end of study feasibility questionnaire, 89% of TRE subjects and 100% of those following a Mediterranean diet would recommend the intervention to others. Long-term follow-up in individuals with Metabolic Syndrome prescribed a TRE diet found approximately 63% of the 19 participants involved were still implementing TRE between 8 and 12 h ~16 months after study cessation [11].

Evidence for the biological, behavioral, psychosocial, and environmental factors affecting adherence to TRE is beginning to emerge within the literature [26,29,30]. Qualitative data have highlighted social activities, family life, and work commitments as barriers to TRE [26,31,32]. In a pilot study intervention, the authors reported participants had difficulty in adhering to the restriction every day, indicating social events influenced diversion from the protocol, although participants did suggest that demonstrable health benefits and the enablement of a flexible approach would incentivise them to consider TRE in the future [5].

A questionnaire-based study analyzing the proof-of-concept of the current TRE protocol symmetrically restricting the daily eating window by 3 h found ~59% of participants with an eating window ≥12 h could feasibly incorporate the restricted eating pattern into their daily routine [29]. The data also demonstrated that factors influencing motivation to undertake TRE included cost, time availability, and health benefits on workdays, as well as wake time, bedtime, time availability, motivation to change, and health benefits on free days. Despite the research suggesting TRE is relatively adherable, caution must be applied when interpreting these findings, since the volunteers were likely to be motivated to participate and comply with TRE regulations.

The studies described above were all conducted over a short timeframe between 4 and 24 weeks. Further, most studies have used self-reported diet diaries to monitor adherence, and these are subject to misreporting bias. Further research is needed to objectively assess adherence to TRE regimes, identify factors that may impact adherence, and explore preferences relating to the proposed TRE or other protocols.

Therefore, the present TRE study incorporated a 3 h symmetrical reduction to habitual eating windows over 12 h by delaying and advancing first and last daily eating occasions by 1.5 h, respectively, with the aim and objective of: (i) exploring objective and subjective adherence to the protocol during 5 weeks of TRE; (ii) exploring the preference for the current TRE protocol and prospective use with a focus on the positives and negatives, and (iii) identifying the barriers to maintaining TRE.

## 2. Materials and Methods

### 2.1. Study Design

**Figure 1:**
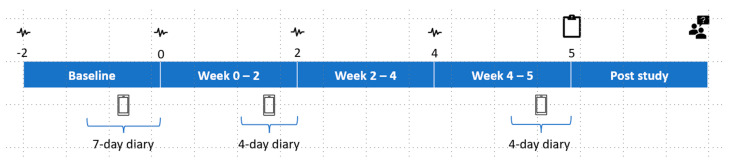
Study design and timeline of procedures. Continuous glucose monitors as indicated by the symbol 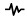 were changed every two weeks beginning at the start of baseline (Week −2) until the beginning of Week 4. Data collected at home included a diet diary 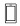 on three separate occasions (Week −2–0, Week 0–2, and Week 4–5). End of study data collected included an exit questionnaire 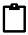 (Week 5) and exit interview 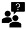 (Post study).

In this 7-week TRE study, participants were assigned to either the TRE (intervention) or control group using age, weight, and BMI to ensure that demographics were equivalent between groups. The study consisted of a 2-week baseline period followed by 5-weeks of control or TRE. The intervention length of 5-weeks was designed so that adherence could be reviewed and compared at three phases of the trial (during baseline, during 2 weeks at the start of TRE, and 3 weeks in the final phase of TRE). The main purpose of this was to explore whether rates of adherence varied depending on the phase of the intervention. The intervention phase was deemed long enough to provide sufficient experience of following TRE under free-living conditions and to identify the barriers that impact on the ability to maintain adherence. An outline of the study and a timeline of procedures are provided in Figure 1.

In total, there were 6 study visits to the Clinical Investigation Unit (CIU) at the University of Surrey, including screening, start of baseline, end of baseline, and after 2 weeks, 4 weeks and 5 weeks of intervention. Participants attended study visits at the end of baseline, and 2 weeks and 5 weeks of intervention in a fasted state (≥12 h). The study received a favorable ethical opinion from the University of Surrey ethics committee: UEC 2019 064 FHMS. This study was conducted in accordance with the ethical principles expressed in the Declaration of Helsinki. Recruitment began in January 2022, and participant enrolment started on 31 January 2022 and ended on 1 May 2022. The study ended on the 19 June 2022.

#### 2.1.1. Study Intervention

At baseline, participants were instructed to maintain their habitual eating times, then either reduce the eating window by 3 h for the intervention phase (TRE group) or continue with routine eating times (control group). To reduce any potential bias towards early or evening eating, participants were instructed to delay and advance their first and last energy intake times by 1.5 h each in accordance with averaged eating time data recorded at baseline. Participants in both groups were allowed to eat ad libitum within their habitual or adjusted eating windows. Participants could consume water, tea and coffee without milk and sugar, sugar-free herbal teas, and sugar-free soft drinks during the fasting period. Under both conditions, participants were given a ±30 min timeframe in which they could adjust their eating window but were encouraged to maintain their routine as consistently as possible.

#### 2.1.2. Participants

**Figure 2:**
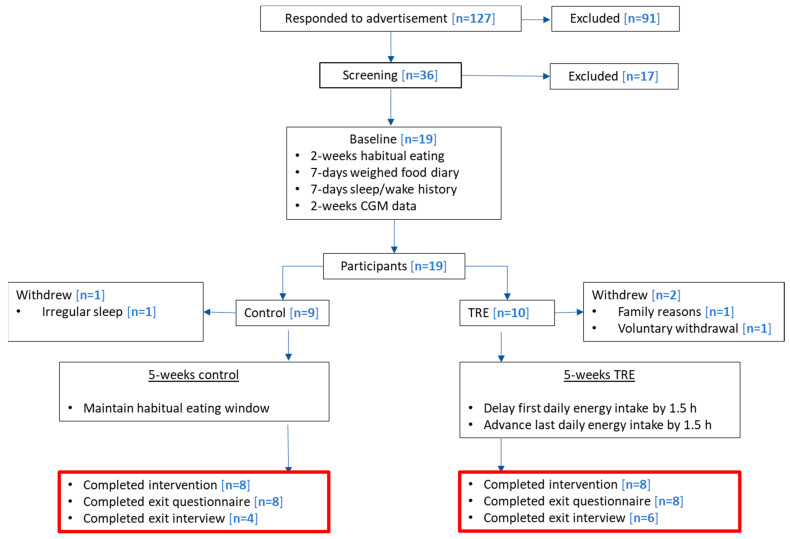
Time-restricted eating study CONSORT diagram. The number of participants from the TRE and control groups completing the study, exit questionnaire, and exit interview are presented in the text boxes with a red border.

Non-obese (BMI 18.5–29.9 kg/m^2^) males and females (aged 18–50 y) with normotensive blood pressure (<140/90 mm Hg) and an eating window ≥12 h were recruited to take part in this TRE trial. Potential participants were excluded if any of the following were reported: chronic illness, regular medication affecting appetite or weight, history of shift work, weight change of ±3 kg in the last 6 months, regular smoking, travel across ≥2 time zones one month prior to or during the trial, and current involvement in other clinical trials. After dropouts, *n* = 16 participants met the intervention (*n* = 8) and control (*n* = 8) conditions; *n* = 16 completed the exit questionnaire and *n* = 10 completed the exit interviews. An outline of the study consort is displayed in Figure 2.

### 2.2. Adherence

Adherence was assessed using data from the time-stamped dietary assessment, the CGM monitors and data from the exit questionnaire and exit interviews.

#### 2.2.1. Time-Stamped Dietary Assessment

Dietary intake was recorded using the Libro app (Libro, v.8, Dublin, Nutritics, 2019) available for both iOS and Android smartphones and automatically uploaded to Nutritics software for analysis (Nutritics, Research Edition, v5.64, Dublin, Nutritics, 2019). Participants recorded 7 days of weighed food intake [33] in the last week of the baseline phase to establish the mean time of first and last eating occasions and overall eating window, from which a delayed eating start time, and advanced eating end time and overall eating window, were calculated. During the intervention period, participants in both groups recorded 4 days of dietary intake in the week leading up to visits at 2 weeks and 5 weeks of the investigation. Participants were provided with a Libro app user manual and individual training support on how to accurately categorize eating occasions within the app. Each food item was entered into the appropriate category as breakfast, lunch, dinner, snack, or drink within the Libro app, along with the quantity, and time of consumption. Food items could be found in the app by utilizing the search engine or by scanning the barcode of food packaging, if applicable. Certain foods featured picture guides to help with estimating portion size, and participants could upload and save the nutritional content of items not available on the Libro app database.

#### 2.2.2. Continuous Glucose Monitors (CGM)

Adherence to the TRE and control group instructions was monitored by comparing CGM data to the time-stamped diet diaries. Freestyle Libre Pro iQ (Abbott, Wiesbaden, Germany) continuous glucose monitoring (CGM) was used throughout the study to observe 24 h glucose responses in accordance with recorded dietary information. The sensor was inserted into the back of the upper non-dominant arm and scanned to confirm data were ready to record. Participants returned every two weeks to have a new CGM sensor attached. Due to sensor error or poor application, some glucose data are reported as missing.

#### 2.2.3. Scoring Adherence

Objective scores for the rates of adherence to the TRE protocol were agreed by the research team. Adherence was evaluated using CGM data according to whether they met the following criteria: (i) the eating window after baseline was reduced by 3 h and was ≤12 h in total; (ii) first and last eating times were delayed and advanced by 1.5 h in accordance to baseline mean times; (iii) glucose increments began ±30 min within the self-reported first and last eating times. Controls were considered non-adherent if the reported eating window was <12 h. The first and last daily eating occasions were identified when glucose levels were elevated by ≥1 mmol/L for ≥1 h or longer.

**Figure 3:**
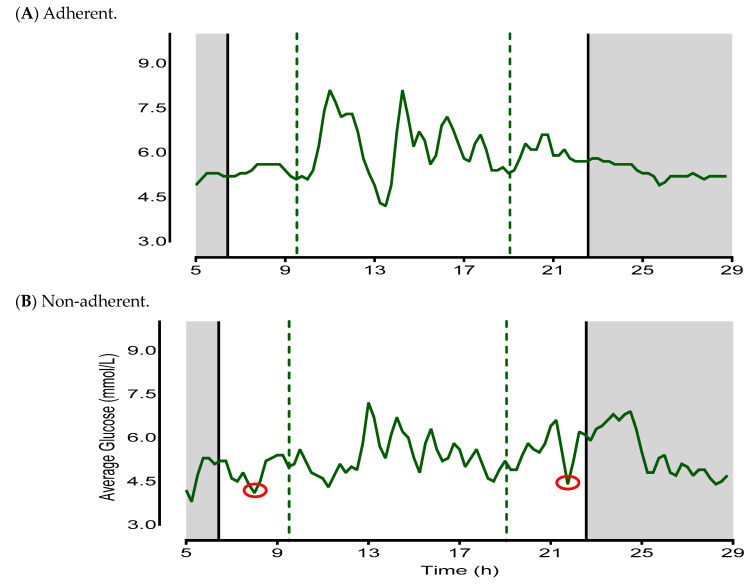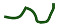 = glucose levels; 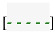 = mean delayed and advanced eating occasions; 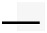 = mean sleep and wake times; 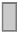 = sleep phase; 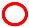 = suspected energy intake outside of reported eating window. (**A**) is an example of an adherent day with the CGM response in close correspondence to the reported mean first and last eating occasion, and the overall eating window is <12 h. (**B**) is an example of a non-adherent day where CGM responses appear before and after the reported mean first and last eating occasions of the day, and the overall eating window is >12 h.

An example of how adherence and non-adherence was calculated is demonstrated in Figure 3. CGM data were analyzed in accordance with time-stamped dietary assessments, providing an estimate of the mean first and last eating times and subsequent eating window.

Adherence was coded as Yes/No for each 24 h period in an Excel spreadsheet. The percentage of adherence was calculated as: (number of days adherent ÷ number of intervention days, i.e., 35 days) × 100. When CGM data were missing, however, the number of available intervention days was recalculated to determine the number of days adherent.

#### 2.2.4. Exit Questionnaire

The exit questionnaire aimed to explore and quantify subjective rates of adherence to the protocol. For the TRE group, the questionnaire was designed to investigate perceived adherence to the 1.5 h morning delay and evening advance in eating times, and overall compliance with the 3 h reduction in habitual eating windows. For the control group, the exit questionnaire aimed to investigate their anticipated adherence if they had been selected to take part in the TRE group. An example of the questionnaires and the Likert scale responses used for both groups can be found below.

Participants assigned to the TRE group responded to the following questions to mark their perception of adherence: (i) How did you find the TRE intervention? (Extremely difficult/Somewhat difficult/neither easy nor difficult/Somewhat easy/Extremely easy); (ii) On how many days of the week were you able to comply with the 1.5 h delay to the timing of first energy intake in the morning? (Never 0 days/Sometimes 1–2 days/Approx. half 3–4 days/Most of the time 5–6 days/Always 7 days); (iii) On how many days of the week were you able to comply with the 1.5 h advance to the timing of last energy intake in the evening? (Never 0 days/Sometimes 1–2 days/Approx. half 3–4 days/Most of the time 5–6 days/Always 7 days); (iv) Were you able to reduce your eating window by 3 h overall? (Never 0 days/Sometimes 1–2 days/Approx. half 3–4 days/Most of the time 5–6 days/Always 7 days).

For the control group, participants were asked to estimate their level of adherence if they were assigned to follow the TRE intervention using the same response frames as the TRE group: (i) How do you think you would find the TRE intervention? (ii) On how many days of the week do you think you would be able to comply with the 1.5 h delay to the timing of first energy intake in the morning? (iii) On how many days of the week do you think you would be able to comply with the 1.5 h advance in the timing of last energy intake in the evening? (iv) Do you think you would be able to reduce your eating window by 3 h overall?

#### 2.2.5. Exit Interviews

In a semi-structured interview format, participants were asked to give feedback on their overall experience and attitudes towards following TRE. Participants in the TRE group were asked to provide an assessment of their adherence to the morning delay and evening advance in the timing of energy intake. Participants were encouraged to reflect on the factors that either enabled or restricted them from adhering to TRE, along with comments on their overall level of adherence to the protocol. Participants from the control group were asked these questions hypothetically to investigate their perceived likelihood of adherence to TRE. An overview of the exit interview questions can be found in Table 1. Full transcripts are available as Supplementary Materials.

### 2.3. Preference towards TRE

Participant preference for TRE was assessed through the responses to the exit questionnaire.

#### Exit Questionnaire

The exit questionnaire aimed to review the preference for TRE in participants from both groups. The questionnaire investigated post-study plans to follow TRE, how many days participants can realistically follow TRE, and the duration of restriction that could reasonably be adhered to. The questionnaires attributed to measuring the preferences towards TRE and the answers available on a Likert scale can be found below.

TRE group participants were asked the following questions to explore preference for TRE: (i) Do you plan to continue with following TRE? (Never 0 days/Sometimes 1–2 days/Approx. half 3–4 days/Most of the time 5–6 days/Always 7 days); (ii) Realistically, how many days a week do you think you could follow TRE on? (0/1/2/3/4/5/6/7); (iii) Please select the duration of time that you could consistently adhere to in terms of restriction to your daily eating window. (<0.5 h/0.5–1 h/1.1–2 h/2.1–3 h/3.1–4 h/>4 h).

Control group participants were asked the following questions to explore preference for TRE: (i) How many days a week would you consider following TRE? (Never 0 days/Sometimes 1–2 days/Approx. half 3–4 days/Most of the time 5–6 days/Always 7 days); (ii) Realistically, how many days a week do you think you could follow TRE on? (0/1/2/3/4/5/6/7); (iii) Please select the duration of time that you could consistently adhere to in terms of restriction to your daily eating window. (<0.5 h/0.5–1 h/1.1–2 h/2.1–3 h/3.1–4 h/>4 h).

### 2.4. Statistical and Thematic Analysis

Statistical analysis was undertaken using SPSS (version 28; IBM Corp., New York, NY, USA). Exit questionnaire responses were analyzed using an independent samples *t*-test to review any differences in subjective and perceived rates of adherence to and preferences for TRE between both groups. A chi-squared analysis was run to test the distribution of sex ratio between groups. A two-way repeated measures ANOVA was carried out to observe the effects of TRE by time, by group, and by time and group interaction on changes to first and last eating occasions and duration of the eating window. A Bonferroni test was carried out to identify in which group and between which different time points of the intervention did a significant change occur.

Interviews were recorded and transcribed in Microsoft Teams and were edited post-interview using Microsoft Word. The transcriptions were thematically analyzed to identify the key factors describing the experienced and anticipated positives and negatives of following TRE. In addition, participants from both groups identified some of the experienced and anticipated barriers to, and incentives towards, TRE.

## 3. Results

### 3.1. Participants

In total, 16 participants completed the intervention and were included in the analysis (15 females = 93.8%; 1 male = 6.2%). An overview of the group characteristics can be found in Table 2. At the beginning of the study there were no significant differences between the groups regarding sex, age, weight, or BMI. Most participants described themselves as white (*n* = 11; 68%), Asian British (*n* = 3; 18.8%), black, black British, Caribbean or African (*n* = 1; 6.3%), or Middle Eastern (*n* = 1; 6.3%). Half of the participants were aged between 19 and 23 years old (*n* = 8; 50%). The remaining participants were aged between 25 and 46 years old (*n* = 8; 50%).

### 3.2. Adherence

#### 3.2.1. Time-Stamped Dietary Assessment

**Figure 4:**
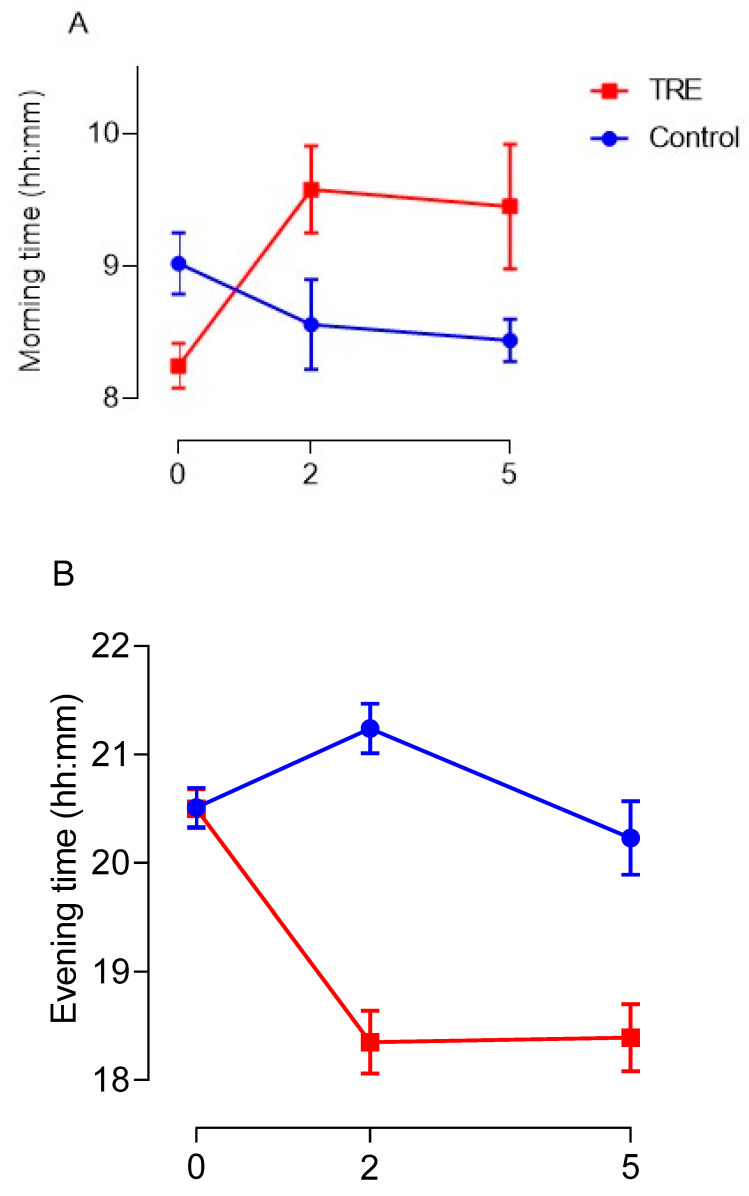

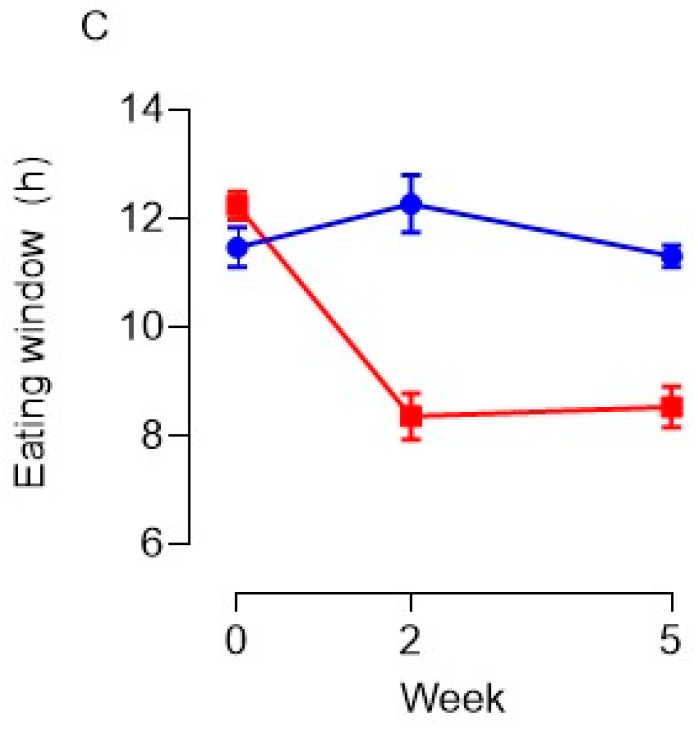
Review of the impact TRE had on changes to the time of first energy intake in the morning (**A**), last energy intake in the evening (**B**), and alteration to the overall eating window (**C**). Data are given as means and vertical bars represent SEM. Blue lines correspond to the control group and the red dash lines highlight the TRE groups’ responses.

Adherence to the protocol was first analyzed using the self-reported time-stamped dietary assessment to estimate the average time of first and last energy intake of the day, and overall eating window at baseline and during the intervention. Figure 4 below shows the self-reported changes made to eating times and eating window between the baseline and intervention phases. A two-way repeated measures ANOVA indicated a non-significant trend towards TRE in the intervention group after the baseline phase delaying first energy intake in the morning (Figure 4A) (*p* = 0.065). A post-hoc Bonferroni test revealed there was a significant delay to the timing of first energy intake in the morning between the end of baseline and after 2 weeks of intervention (*p* = 0.025) in the TRE group; however, the delay to morning eating time between baseline and week 5 of intervention was non-significant (*p* = 0.092). Overall, TRE in the intervention group had a significant effect on advancing the time of last energy intake in the evening (Figure 4B) (*p* < 0.001) and reducing the overall eating window between groups (Figure 4C) (*p* < 0.001). Specifically, post hoc analysis identified a significant difference to the timing of last energy intake of the day between the end of baseline and after 2 weeks of intervention (*p* < 0.001) and baseline and week 5 of intervention (*p* = 0.006) in the TRE group. The overall eating window in the TRE group was significantly reduced after the baseline phase at 2 weeks (*p* < 0.001) and 5 weeks of intervention (*p* < 0.001). A summary table with average eating times can be found in Supplementary File S1.2.

#### 3.2.2. Continuous Glucose Monitoring (CGM)

Table 3 provides a summary of estimated adherence to the protocol using objective CGM data. Adherence was estimated by observing each 24 h CGM recording, noting whether first and last glucose excursions were within ±30 min of the agreed first and last eating times reported in the time-stamped dietary assessments. CGM data were compared alongside time-stamped dietary assessments to review rates of adherence to self-reported first and last eating occasions during baseline and intervention. There are CGM data missing for TRE (*n* = 1) and control group participants (*n* = 3).

The use of CGM devices did not reveal any significant difference between groups in rates of adherence by time, group, or group × time effect, despite lower rates of adherence recorded at 2 weeks and 5 weeks in the TRE group. Based on the adherence rates listed in Table 3, participant adherence in the TRE group was 5.2 ± 1.5 days per week during baseline (75.3%), 4.2 ± 1.5 days per week during 2 weeks of TRE (60.1%), and 4.5 ± 1.7 days per week during the final 3 weeks of the intervention (65.2%). When the intervention phase rates are combined, average adherence over the course of 5 weeks is approximately ~63%. In the control group, adherence during baseline was 5.8 ± 0.3 days per week (82.7%), 5.7 ± 1.4 days per week for two weeks (82.4%), and 5.2 ± 0.4 days per week in the last 3 weeks of the control phase (75.2%). Overall adherence in the control group was estimated to be ~79%.

#### 3.2.3. Exit Questionnaires

The data measuring adherence from the exit questionnaires are summarized in Table 4. Participants assessed their ease or difficulty in following the intervention, along with estimating the number of days adherent to the protocol. TRE participants responded with regard to their experience of following TRE, whereas the control group provided an anticipatory response should they have been following TRE.

Although not a direct comparison, the results in Table 4 demonstrate there was a significant difference between the subjective and prospective rates of adherence to the morning delay, evening advance, and overall 3 h window reduction between TRE and control group participants. The results may indicate TRE is easier to adhere to than is anticipated. Both groups reported they found, or would find, the intervention neither easy nor difficult to adhere to. For both groups, the results indicate the morning delay was or would be easier to adhere to than the evening advance to energy intake (morning TRE group 4.9 ± 0.4 days per week and control group 3.4 ± 0.9 days per week; evening TRE group 4.1 ± 0.6 days per week and control group 2.8 ± 0.7 days per week). Based on the findings in Table 4, TRE participants were adherent to the overall 3 h reduction in habitual eating window on 4.3 ± 0.7 days per week (61%). The control group anticipated they would be able to adhere to a 3 h restriction to habitual eating windows on 3 ± 0.5 days per week (43%). These results highlight TRE participants were able to adhere to the protocol on more days than the control group perceived would be feasible. The outcomes may also suggest that TRE is easier to adhere to than is anticipated.

#### 3.2.4. Exit Interviews

A total of 10 participants (TRE *n* = 6, control *n* = 4) took part in the exit interviews expressing their views on TRE, adherence to the protocol, the positives and negatives of following TRE, their plans for future adherence, and the incentives or barriers to adopting TRE into their daily routines.

##### Summary of Adherence Themes

The majority of TRE participants found the morning delay easier to adhere to (*n* = 5). Factors that helped participants to adapt to the morning delay included taking breakfast to work (*n* = 1), working from home (*n* = 1), and not having the desire to eat in the morning (*n* = 1). Two participants found delaying morning energy intake challenging because they required energy to start their day (*n* = 1), and because of lectures in the morning (*n* = 1). Those who found it easier to advance in the evening stated this was because they cut out late-night snacking (*n* = 1), or they were able to eat an evening meal with their children (*n* = 1). Two of the participants in the control group said they would find the morning delay easy, but due to habitual snacking in the evening, they envisaged restricting this could be problematic. A control group participant indicated they would find it difficult to advance the last energy intake in the evening due to socializing or work commitments.

When the data were analyzed collectively, reports indicated partial adherence to the protocol over the 5-week intervention. There are indications of a preference towards greater adherence to morning delayed eating in comparison to evening energy restriction, which warrants further exploration. Thematic analysis has identified some of the mitigating factors impacting the uptake of daily TRE, and may prove useful for the development of future studies. An overall summarization of participant experiences following TRE, including the positives and negatives, incentives for and barriers to TRE, and any other additional comments, is listed in Table 5.

The feedback from participants provides an insightful synopsis of the key influential factors due for consideration to help develop appropriate TRE protocols that are applicable under free-living conditions.

### 3.3. Preference towards TRE

#### 3.3.1. Exit Questionnaire

The data in Table 6 provide a comparative assessment of participants from both groups and their preference for TRE. The table summarizes the overall number of days for which continuing with TRE was planned, the number of days for which TRE can feasibly be followed, and the amount of time the eating window could realistically be reduced by.

An independent samples-*t* test was conducted to compare preference for TRE between the intervention and control group subjects. There was no statistical difference when analyzing for preference for TRE, including plans to follow TRE, number of days for which it would be possible to follow TRE, and the number of hours participants can realistically reduce their eating window by between the two groups. As regards the TRE group, wherein participants had followed the intervention, the findings in Table 6 show that the number of planned TRE days is fewer than the number of realistic adherable days. Reported restrictions of 3.3 h in the control group and 3.6 h in the TRE group suggest the habitual eating window may be further reduced by ~16–36 h more than the 3 h restriction implemented in this current study, and therefore, a reduction of 3–4 h may be appropriate for future studies.

Overall, the data indicates moderate preference for TRE. The number of estimated adherable days exceeds the number of actual days of planned adherence, which may point to a lack of preference for TRE. There are demonstrable factors affecting the preference for TRE, and this information can be used to develop future TRE protocols.

##### Summary of Preference Themes

Encouragingly, a few participants (*n* = 3) stated they had tried to continue with following TRE, although two did concede that family visiting and work schedules had impacted their ability to adhere. Most of the participants stated that they would prefer to follow TRE during the weekdays, and then have flexibility over the weekends (*n* = 7). Two participants concluded they would find it easier to implement TRE on the weekends because they have better flexibility then, as opposed to during the working week. If the participants were to follow TRE again, most participants said they would prefer to delay the first eating occasion so as to enable greater flexibility in eating in the evening (*n* = 7). Just two participants stated they would prefer to advance the last energy intake of the day. One participant reported they would not be inclined to ever follow TRE because they do not want to be restricted with when they eat, and would prefer to use their own self-awareness to determine when they eat.

##### Positives of TRE

TRE was reported as being easy to follow (*n* = 4), with one participant benefiting from the structure of routine it provided. Improvements to sleep quality (*n* = 2), better digestion (*n* = 1), reduced bloating in the morning (*n* = 1), maintaining hydration (*n* = 1) and reports of having more energy in the morning were some of the positives of following TRE. Several participants from both groups described how taking part in the study made them consider their overall dietary quality, and influenced their eating habits by reducing their intake of highly palatable foods (*n* = 3).

##### Negatives of TRE

There were commonalities amongst the participants as regards the challenges with following TRE, including social events (*n* = 5), work commitments (*n* = 3), evening leisure activities (*n* = 1), living arrangements (*n* = 1) and family life (*n* = 1). Another negative was the amount of planning that adhering to TRE required, as regards the consideration of when their routine enabled them to eat throughout the day, and whether this aligned with their desire to eat (*n* = 3). A lack of flexibility, modifications to eating behavior, fatigue, and unpredictable shift patterns resulting in skipped meals or snacking on highly palatable foods were highlighted as some of the negatives of following TRE.

##### Incentives of TRE

The main incentive that participants shared was for induced weight loss (*n* = 4) or the prevention of unwanted weight gain (*n* = 2). Participants from both groups described how the possibility of improved sleep would motivate them to take up TRE (*n* = 3). Glucose regulation (*n* = 1), benefits to gut health (*n* = 1) and mental health (*n* = 2), reduced alcohol intake in the evening (*n* = 1), and the avoidance of unhealthy foods (*n* = 2) also incentivized participants to follow TRE.

##### Barriers to TRE

The factors acting as the greatest barriers to TRE uptake were work commitments (*n* = 5) and social activities (*n* = 5). One participant identified social media as a negative barrier that would prevent them from adhering to TRE. Specifically, they noted that food advertisements seen in the evening influence their desire to eat, and increase the likelihood of them eating. Living arrangements and, in particular, house sharing with friends were perceived as barriers to TRE, indicating that kitchen availability impacts when they could cook an evening meal (*n* = 2). Interestingly, one participant mentioned how an overly complicated TRE protocol may become a barrier itself if it is hard to follow. A lack of motivation or falling out of routine were also noted as potential barriers to adherence (*n* = 1). Stress and using food as a coping mechanism were also highlighted as potential barriers to maintaining TRE, especially in the evenings. Visiting family was noted as a factor that restricted one participant from continuing with adhering to TRE once the study was over.

## 4. Discussion

This was an exploratory study combining the use of self-reported diet diaries, objective CGM data, subjective questionnaires and qualitative interviews to investigate in depth the rates of adherence to and preference for a TRE protocol. The protocol prescribed to participants in the TRE group consisted of a 3 h reduction in habitual eating windows observed during the baseline phase. This reduction was achieved by delaying the first energy intake and advancing the last energy intake by 1.5 h, respectively.

As suggested by previous TRE studies, group adherence to the protocol appeared to be high, as indicated by a significant reduction in the eating window (Figure 3C). Participants were permitted ±30 min to adjust their eating window to maximize adherence, and self-reported food diaries demonstrate the TRE group were compliant in reducing their eating window by 3 h. More specifically, the morning energy intake in the TRE group was delayed by 1 h 30 min, matching the requirements of the protocol. On the other hand, the last energy intake was advanced by 2 h 17 min, potentially highlighting a preference for restricting eating in the evening that may coincide with previous studies utilizing eTRE regimes [7,17,19,20,21,34]. It is worth noting this increased restriction in the evening contrasts with the qualitative assessment, which indicated most participants found the morning delay easier to adhere to and would prefer to delay morning energy intake. Reporting bias may have influenced the inclusion and accuracy of self-reporting dietary behaviors, particularly in the evening. Self-reported eating times suggest that the TRE group’s eating window was reduced on average by 3 h 47 min during the 5-week study. The earlier advance in the timing of evening energy intake might imply that time of day flexibility is a prerequisite of TRE if it is to be used as a practical dietary approach in the free-living environment. So-called early TRE (eTRE) (08:00–13:30 [35]; 07:00–15:00 [34,36]; 08:00–14:00 [17,20]; 08:00–16:00 [19]; 08:00–17:00 [18]) and delayed TRE (dTRE) (12:00–20:00 [26,37]; 12:00–21:00 [18]; 13:00–20:00 [12]) protocols have been utilized in several studies, although preference for any of these programmes has not been assessed. Based on the results implying preference for delaying the first energy intake of the day, future research would need to monitor the effects this would have on postprandial responses to meals later in the daytime and evening in order to clarify whether these would be beneficial or harmful to cardiometabolic health.

The rationale for utilizing CGM devices was based on the objective role they may play in monitoring adherence to an agreed TRE protocol practiced under free-living ad libitum conditions. In this study, time-stamped food diaries recorded at three separate phases of the trial were assessed to establish first and last eating occasions, along with overall eating window. Initially, at baseline, mean first and last eating times calculated from the time-stamped dietary assessments were used to specify the amended first and last eating requirements for the intervention phase. These reported TRE eating times were examined alongside the corresponding CGM data, subsequently suggesting adherence to be in the range of ~60% and ~65% midway through and towards the end of the intervention. Our CGM data provide a novel insight into monitoring adherence to a time-orientated intervention, estimating TRE participants adherence rates to be ~63% compared to ~79% in the control group. This may explain why the anticipated weight loss and reductions in body adiposity were not found, while previous research proposes adherence to TRE should reach >70% in order for it to be effective [28]. Furthermore, the findings of the self-reported dietary assessment may suggest greater adherence when a record of energy intake is required, and CGM devices may be particularly useful in observing deviations from the protocol under free-living conditions.

Our exit questionnaire data corroborate the findings of the CGM devices, estimating overall adherence to TRE to be ~61%. In the TRE group, morning adherence was apparently achieved ~70% of the time, equating to 4.9 d per week. In the evening, adherence was approximately 48% or 3.4 d per week. In combination, these values estimate overall adherence to be ~59%. The data may imply that some of the conditions of TRE are unfeasible, or that some aspects of free-living schedules make adherence difficult to maintain. Our research highlights the reality of adopting TRE into daily routines, and although in theory TRE offers simplicity to modified dieting, adherence is likely to play a significant part in its success.

In the qualitative assessment, participants described the morning delay as being easier to adhere to, whereas some found this a challenge. The interviews also demonstrated that some found advancing the last eating time of the day was made easier by adapting evening eating behavior, or by having dinner with their family. The main positives of TRE were its easily followed instructions, the improved quality of sleep, it provision of a routine, a lowered bloated feeling, and raised awareness of eating habits. The negatives that participants experienced were the impact it had during social and family events discouraging TRE. Delaying morning intake led to feeling fatigued on the commute to work for one participant. Our finding that participants had a preference for practicing TRE during weekdays or weekends corresponds with research reporting time availability to be a considerable determinant predicting the uptake of TRE [29]. One participant working placement shifts at a hospital commented on the influence that their unpredictable schedule had on meeting TRE adherence, citing the omittance of meals or snacking on highly palatable foods as a way to comply with the TRE protocol. Future TRE investigations need to weigh up the benefits and consequences of intentionally foregoing meals, or relying on energy-dense foods to aid adherence to TRE.

A number of key factors, including weight loss or weight maintenance, improved sleep quality, glucose regulation, effects on gut health and mental health benefits, and indirect changes to diet quality incentivized participants to take up TRE, echoing the findings of previous qualitative research [30]. Despite weight loss being the main prerequisite incentivized the uptake of TRE amongst participants, our research clearly demonstrates there are other key motivating factors that could make TRE an appealing strategy for individuals living both with and without overweight and obesity.

Similar to previous research [38], here, the barriers to TRE adherence included work and social conflicts, and daily routines [32,39]. A series of unique challenges were raised that affected adherence, including advertisement of desirable foods, house sharing limiting the time available for preparing meals, the need to plan mealtimes, and holiday or cultural factors that favor late-night eating. This study therefore adds to the literature by providing further insight into the positives and negatives participants face when committed to TRE, along with potential barriers to and incentives for a TRE dietary pattern.

Our quantitative assessment of preference aimed to establish the number of days and hours for which participants from both groups would be willing to implement TRE post-study. The exit questionnaire data suggest that the TRE and control group participants plan to follow TRE on 3 days and 3.4 days per week, or ~43% and ~49% of the time, respectively. Realistically, however, the intervention group participants that experienced TRE reported they could adhere to a self-selected protocol on 4.6 days per week, or ~66% of the time. The controls felt they could realistically incorporate TRE into their daily routine on 3.6 days per week, or ~51% of the week. The duration of restriction was similar between both groups, with the TRE group stating a 3.6 h reduction and the controls suggesting they could reduce their eating window by 3.3 h per day. What remains unknown is the long-term feasibility of implementing these suggested eating regimes. Further research with larger samples sizes is required to better understand the rate of adherence and the achievement of targeted health benefits, which can be used to inform personalized TRE protocols.

### Strengths and Limitations

This study has a number of strengths. First, our study delivers an objective assessment of adherence to TRE using CGM data. The inclusion of a mobile phone-based app enabling the recording of time-stamped energy intake provides a measure of timed food consumption, with the assumption that subjects are observant when recording data. CGM data (compared with self-reported food diaries) may be useful in identifying adherence to TRE. Our simple exit questionnaire has generated a wealth of quantitative data on self-reported adherence to the morning and evening constraints, and overall adherence to the 3 h reduction in the eating window. Our qualitative approach to exploring participant experiences helped us accumulate useful insights into the strengths and limitations of TRE, along with identifying some of the novel barriers and incentives that would motivate individuals to take up TRE.

The limitations of the study included its symmetrical restriction of the eating window, which negates some of the preferences participants may have for morning or evening eating. Future research is required to ascertain to what extent preference towards early or delayed TRE are driven by chronotype. The small sample size and short study duration are noteworthy limitations of the study. Moreover, the small timeframes used to capture dietary intake over 7 days at baseline or 4 days during two separate intervention stages may not accurately reflect the dietary habits of the participants. Although CGM data provide a useful insight into glucose excursions in relation to dietary intake, some physiological responses may have gone undetected in response to low-carbohydrate meals or snacks. Furthermore, some other excursions may have been undertaken as a physiological response to waking [40]. The number of participants taking part in the qualitative section of the section was low, and therefore, we were only able to collect the views of a small section of the cohort involved. The mixed responses regarding preference for early or delayed TRE highlight the limitations of our study in symmetrically restricting the eating window, and emphasize the importance of adopting a personalized approach to TRE scheduling [6].

## 5. Conclusions

Self-reported food diaries may provide a useful account of eating times, but accuracy in reporting is crucial for this to be successful. CGM devices used in cohesion with self-reported diaries may help with accurately reviewing adherence to TRE regimes with specified eating and fasting times. Future studies with larger sample sizes and longer intervention periods are required to investigate the effects of a self-selected TRE regime on rates of adherence.

Despite being proposed as a simplified dietary approach, adherence to TRE is affected by lifestyle and behavioral factors. Overcoming these barriers to adopting TRE warrants a personalized approach in order to successfully incorporate TRE in relation to scheduling, social and family activities. The flexible scheduling of TRE is crucial to enabling individuals to self-select an eating window, along with a start and end time to eating occasions, which will maximize the likelihood of sustaining adherence.

## Figures and Tables

**Table 1:** Exit interview questions exploring adherence to TRE.

TRE Group Experience of TRE	Control Group Attitude towards TRE
How did you find following the TRE intervention? Why was that?	How do you think you would find following the TRE intervention? Why is that?
What were the negatives of following TRE? What were the positives of following TRE?	What do you think the negatives of following TRE would be? What do you think the positives of following TRE would be?
Did it affect your day-to-day living in any way? Did it affect your food choices in any way? Did it affect your sleep in any way?	Do you think it would affect your day-to-day living in any way? Do you think it would affect your food choices in any way? Do you think it would affect your sleep in any way?
Adherence	Adherence
On how many days of the week were you able to comply with the 1.5 h delay to the timing of first energy intake in the morning? What enabled you to achieve this? What restricted you from achieving this?	On how many days of the week do you think you would be able to comply with the 1.5 h delay to the timing of first energy intake in the morning? What would enable you to achieve this? What would restrict you from achieving this?
On how many days of the week were you able to comply with the 1.5 h advance to the timing of last energy intake in the evening? What enabled you to achieve this? What restricted you from achieving this?	On how many days of the week do you think you would be able to comply with the 1.5 h advance to the timing of last energy intake in the evening? What would enable you to achieve this? What would restrict you from achieving this?
Were you able to reduce your eating window by 3 h overall by choosing your own feeding window? What did you do?	Do you think you would be able to reduce your eating window by 3 h overall if you were able to choose your own feeding restriction? What would this look like?

**Table 2:** Participant characteristics (*n* = 16).

	TRE (*n* = 8)	Control (*n* = 8)	*p*
Sex (F:M)	8:0	7:1	0.351
Age (y)	28 ± 9.5	27.4 ± 8.1	0.889
Weight (kg)	66.4 ± 10.1	66.6 ± 10.1	0.970
BMI (kg/m^2^)	24.1 ± 3.0	23.0 ± 1.6	0.435

Note: Data displayed are the mean and standard error of the mean SEM. A chi-squared analysis revealed no significant difference in the distribution of sexes between groups at baseline. There were no statistical differences in mean age, weight or BMI of both groups based on outcomes of an independent samples *t*-test.

**Table 3:** Summary of adherence from CGM data.

TRE *n* = 7					Control *n* = 5				Time	Group	Group × Time
	W0	W2	W5	Mean	W0	W2	W5	Mean	*p*	*p*	*p*
Adherence (%)	75.3 (7.1)	60.1 (8.3)	65.2 (8.0)	62.7 (8.2)	82.7 (2.6)	82.4 (8.9)	75.2 (2.7)	78.8 (5.8)	0.400	0.176	0.435

Note: Data represent mean adherence % and SEM for time effect, group effect, and group × time effect of condition analyzed using two-way repeated measures ANOVA. W0 is the rate of adherence recorded during the last week of baseline. W2 and W5 represent adherence rates recorded after 2 weeks and 5 weeks of intervention or control.

**Table 4:** Between-group analysis of adherence to TRE.

		Mean ± SEM	*t*	*p*
TRE	How did you find the TRE intervention?	3.1 ± 1.0	0.266	0.794
Control	How do you think you would find the TRE intervention?	3.3 ± 0.9		
TRE	On how many days of the week were you able to comply with the 1.5 h delay to the timing of first energy intake in the morning?	4.9 ± 0.4	−4.320	<0.001
Control	On how many days of the week do you think you would be able to comply with the 1.5 h delay to the timing of first energy intake in the morning?	3.4 ± 0.9		
TRE	On how many days of the week were you able to comply with the 1.5 h advance to the timing of last energy intake in the evening?	4.1 ± 0.6	−4.075	<0.001
Control	On how many days of the week do you think you would be able to comply with the 1.5 h advance to the timing of last energy intake in the evening?	2.8 ± 0.7		
TRE	Were you able to reduce your eating window by 3 h overall?	4.3 ± 0.7	−3.989	<0.001
Control	Do you think you would be able to reduce your eating window by 3 h overall?	3.0 ± 0.5		

Note: Data are reported as the mean and SEM. Mean values relate to exit questionnaire responses recorded on a Likert scale.

**Table 5:** Overall assessment of experiences with TRE.

Area of Investigation	Summary of Thematic Analysis	TRE	Control
Experience of TRE	Challenging to fit in around work and children	☒	☒
	Greater compliance during the week than on weekends	☒	☒
	When to eat required planning around work and social activities	☒	☐
	Habitual breakfast-eaters found delaying morning intake a challenge	☒	☐
	Morning delay was easy for most	☒	☒
	Evening advance was difficult initially for habitual snackers	☒	☒
	Difficult to adhere to on holiday or with family in cultures favoring later eating	☒	☐
	Encouraged increased fluid intake during the fasting phase	☒	☐
Positives of TRE	Easy to follow	☒	☒
	Provided a routine	☒	☐
	Improved quality of sleep	☒	☐
	Feeling better, less bloated, better digestion	☒	☒
	Participants became more conscious of their eating choices	☒	☒
Negatives of TRE	Impacted on social events	☒	☒
	Fatigue and low energy when delaying breakfast	☒	☐
	Difficult to implement at weekends	☒	☐
	Unable to sleep when hungry	☐	☒
Incentives to follow TRE	Improve sleep conditions	☒	☒
	To help maintain weight, lose weight, or reduce abdominal fat	☒	☒
	Improvements to gut health	☒	☐
	Enabling flexibility to the restriction	☒	☐
	Benefits to mental health, mood, concentration	☒	☒
Barriers to following TRE	Social life, living accommodation, social media posts	☒	☒
	Work commitments and lifestyle, activities	☒	☒
	Family routines	☒	☐
	Requires planning to time meals appropriately	☒	☒
	Holidays or cultures with preference for late-night eating	☒	☐
Preference towards TRE	Weekdays on, free days off	☒	☒
	Free days on, weekdays off	☒	☐
Additional comments	Some reported adopting TRE into their daily routine post-study	☒	☒
	Requires discipline to adhere	☒	☐

Note: An overview of the themes related to TRE outlined from thematic analysis. An x represents feedback from 1 or more individuals from that group.

**Table 6:** Comparing preference towards TRE between intervention and control group.

	TRE *n* = 8	Control *n* = 8	*t*	*p*
How many days a week do you plan to continue following TRE on?	3.0 (0.9)	3.4 (0.9)	0.814	0.429
Realistically, how many days per week do you think you could follow TRE on?	4.6 (1.5)	3.6 (0.1)	−1.372	0.192
Please select the duration of time you could consistently adhere to in terms of restricting daily eating window	3.6 (0.7)	3.3 (0.7)	−1.033	0.319

Note: Data are displayed as mean and SEM in brackets. Results indicate responses from a Likert scale.

## Data Availability

Data are contained within the article or supplementary material.

## Supplementary Materials

The following supporting information can be downloaded at: https://www.mdpi.com/article/10.3390/nu15102336/s1, File S1: full interview transcript.
