# Supplementary material for: Exploring Rates of Adherence and Barriers to Time-Restricted Eating
Source: Nutrients. 2023 May 16;15(10):2336. doi: 10.3390/nu15102336 (PMC10223820; doi:10.3390/nu15102336)
Supplement: Supplementary file 1 [file nutrients-15-02336-s001.zip › File S1 - Exit Interviews - Edited transcribes.pdf]

TRF study Exit interviews

(1) How did you find following the TRE intervention?

TRF028: "It is not hard, because it is only a 12-h window. So it is quite easy to follow. Just finish my dinner a little bit earlier and trying not to eat something before I go to bed.

How did you find that?

TRF028: " It's a little bit hard to, Because I live with 7 other girls they cook pizzas and fried chicken. They make me a little bit, and it is hard" "Sometimes my friends they invite me to go to the cinema at midnight and ask if you want something to eat or drink, I have to refuse".

Commented [JPW(-SoB&1]: Positive:
Easy to follow
12-h window not difficult

Commented [JPW(-SoB&2]: Negatives:
Living arrangements - With friends cook food to share
Socialising - have to avoid food

TRF038: I was eating between 9 and 6 or as close to that as possible. Before I was eating between 7 and 8 in the evening. I found it a challenge, to fit in around my work and the children. But it was not impossible. And I quite like a challenge, so it was quite interesting for me.

Would you say those were the main negatives of TRF?

TRF038: Erm, yes, yes, yeah, I did not let it rule my life, you know at the weekends, if I went out for dinner at 8 o clock or something like that, but pretty much Monday to Friday I stuck to it. It just needed planning, you had to prepare.

Commented [JPW(-SoB&3]: Overall experience/negatives:
Found it a challenge - difficult to fit in around work and children

TRF097: Overall was ok, fine, but some days in particular it was a bit more difficult to follow. Maybe depending on daily work and the time that you would be at home. Someday maybe I was finishing a bit later from work, by the time I was home, cooking my dinner it was not perfect the time that I should be eating. But overall apart from that it was quite easy to follow.

Would you say those were the main negatives of TRF?

TRF097:I didn't have negative points, just maybe, I don't know if we could be more flexible. I don't know if every day needs to be the same. For example if one day instead of eating 7.30 maximum and 10.30am in the morning, but on one day I have some issues, and I had to eat dinner at 8pm then I can change, add half an hour to the breakfast, I don't know if that's possible. The important thing is just delaying your breakfast for an hour and a half and then your dinner but can you change the time between the days if you have some problems? I think the important thing is to keep the 14 hours.

Would that enable you to follow it then if there was that flexibility?

TRF097: I think so, yeah.

Commented [JPW(-SoB&4]: Overall experience:
Mixed feelings, ok, more difficult to follow on some days - due to work

Negatives:
No negatives but could be more flexible.

TRF008: Personally, I found the morning really easy, like not having anything the morning was absolutely fine because I'd just have a black coffee. The thing I struggled with was the evening side of things because of my schedule it would mean I would have to actually eat quite a lot before the final eating window cut off, so my feeding window became very short because I was happy to do it in the morning but then because of dance in the evenings which is 3 days a week and then I have Korean class 1 day a week as well, so I can eat as close as I want to Korean but that starts at 6.30pm

Commented [JPW(-SoB&5]: Positives:
Easy to follow - Morning easy

Negatives:
Evening hard - due to social life, leisure activity
Requires planning

so I still had to have finished before then because I cant eat in class and with dance I have to eat sooner otherwise I feel sick in class. It just meant that I was, I had to reduce my window even more which mean I basically didn't eat very much which to be honest wasn't that hard, but it just was a bit annoying having to plan and think about having to eat when I'm not necessarily hungry knowing that I'll be hungry but knowing I wont be able to eat then.

Did you find that challenging then?

TRF008: Not really, I mean it was slightly challenging in the sense that we sometimes go out for, normally we go out for drink or food after dance on a Friday when it's in person and everybody in class knew I couldn't eat because id go out with them and be like no I wont have anything and that's quite difficult when food is such a sociable thing. In my day to day life, it wasn't that hard it was just a bit inconvenient.

TRF124: I found it good, for me it wasn't hard to do. Especially delaying the morning energy intake, perhaps because I was used to eating later at night and snacking and eating snacks up until late into the evening. That was probably the hardest part but after a few days it was fine, became normal, and I found it very beneficial for me. I was looking better, I carried on after the study and now I haven't done it for the past 3 weeks and I see the difference.

What differences do you notice?

TRF124: well, I feel now, bloated. Not bloated but late at night, I don't sleep that well, I tend to go to sleep later, and I wake up more tired in the morning and then I noticed a difference in my belly, like when I wake up in the morning my belly would be swollen and when I was doing TRF I was feeling much better.

Commented [JPW(-SoB&6]: Positives:
Easy to follow
Delaying the morning - easy
Looking better, felt better, slept better

Negatives:
Harder to adapt to no snacking at night
Once adjusted it was ok

TRF064: I think it was especially hard for me to follow during the morning time because that is when I get my first energy in the morning, I guess, that was before I walk and start working especially because I have to walk in to campus. I felt as the time went on, the first day wasn't as bad, I mean sometimes I do skip breakfast, I guess my body couldn't get used to it, like just skipping breakfast in the morning until a bit later. And also sometimes there would be some things in the way from having breakfast as soon as 11am hits because that was the middle of the day so I would have to push it back until 12pm and I would have to adjust my feeding window to be 8 hours again. But evening wise, it wasn't that bad, it just meant I was putting off dinner and would just have it a bit earlier. It was just the breakfast, I found it harder to comply or feel as good because that would be when I have my coffee.

Commented [JPW(-SoB&7]: Adherence:
Hard to follow in the morning - need energy in the morning to function

Positives:
Evening not bad -

(2) What were the negatives of following TRF?

TRF028: "Most of the time, it wouldn't get in the way of anything".

TRF008: The thing I found hardest to be honest, was just recording all your food, like that is just annoying.

Commented [JPW(-SoB&8]: Negative:
Recording food intake

TRF124:I mean I didn't see any bad points, it's just getting used to it in the beginning, not eating at night that was the hardest point for me, but not bad. And then there was a period when I was eating more sugary things but I think that was related to time, maybe my menstrual cycle. And I cant think of any bad things really, I didn't feel like I was hungry or I couldn't manage my hunger or I wasn't eating enough. And then in a bad way, probably when you have social events that could be a problem, like late at night, that's the only thing that I could think of. I would say no sorry I cant come out for dinner because I'm doing TRF so that's probably only the hard part.

Commented [JPW(-SoB&9]: Positives:
No reported changes in hunger, or not eating enough

Negative:
Social events

TRF064: sometimes on my way to uni I would feel a bit lightheaded and fatigue or maybe it was just more like psychology but towards the end of my walk I'd feel just like so tired, and it would take me a while to get back, not motivated to work, but to get my energy up to work, whereas right now after I've gone back to my original morning routine I feel much more energetic in the morning. Yeah I just feel like I'm used to this just having my first energy intake before I do anything else, rather than do something and then eat. So negatives, if I'm with friends out or when you go to a restaurant people are ready at different times, and I was like no I'm ok I'll stop eating as soon as this time hits, especially in the earlier phase in the TRF I didn't want to sway from the protocol where I was like no I'll stop, but there were some instances with gatherings when dinner would start much later than when I finished eating so in social cases it is harder in the evening. But for me, it was fine, just social gatherings were harder.

Commented [JPW(-SoB&10]: Negatives:
Lightheaded and fatigued when walking
Lack of energy and enthusiasm to start work
Social events

(3) what were the positives of following TRF?

TRF028: "I think my sleep condition got better, because I used to drink some cola or something before I go to bed, and it contains caffeine, so it affected my sleep. I cant sleep well, after TRF I feel much better.

Commented [JPW(-SoB&11]: Positives:
Sleep condition improved - stopped drinking cola before bed.

TRF038: "Well, I mean I think I lost about a kilo which was nice, and I did feel a bit more lean, but apart from that. It made me think twice about what I was eating. So for example, before if I was peckish in the evening, I might have eaten a big bag of crisps and not really thought twice about it, but because I know I was finishing eating at 6, I did not. And I would have like a mint tea instead.

Commented [JPW(-SoB&12]: Positives:
Increased awareness of dietary quality - improved food choices - stopped from eating crisps and opted for a min tea instead.

You first mentioned that you found it good: What did you find good about it?

TRF124: I felt more energised, I felt better physically. Sometimes I'd have stomach aches in the morning, probably because of digesting the night before and when I was doing the TRF I was fine. Sleeping better as I mentioned.

Commented [JPW(-SoB&13]: Positives:
Felt more energetic
No stomach discomfort in the morning
Improved sleep - fell asleep faster and not waking up at night

(4) did it affect your day to day living in any way?

TRF028: "Not really"

TRF097: Not really. I think the only thing I had to do was I had to think about food a bit more. Normally I would just eat when I want and don't think about my plan. But obviously because I did have to think about it a bit more, like when can I finish eating if I start now.

Commented [JPW(-SoB&14]: Overall experience: Had to think about food more - plan when to start and end eating

TRF124: in a better way yeah, in the sense that like I had a routine that I was sticking to, that I was following, and I like routines and I was feeling more energised, and I was paying more attention to what I was doing because I wasn't distracted by food in the evening or the morning.

Commented [JPW(-SoB&15]: Positives:
Provided a routine
Improved concentration by no longer having food as a distraction in the morning and evening

(5) did it affect your food choice in any way?

TRF028: "During the 5-weeeks of intervention I drink less, less soft drinks and no alcohol all the time, and I spent less money on snacks like chips".

Commented [JPW(-SoB&16]: Positives:
Encouraged to drink less soft drinks and alcohol
Saved money on snacks

TRF124: no actually, because I felt like I was doing something positive for myself. Not that I knew much about TRF but there's a lot going round now, like with alternate day fasting, so I actually started eating better I think. Like it motivated me to eat better, that's the only way that it affected it.

Commented [JPW(-SoB&17]: Positives:
Motivated to eat better

TRF064: erm no, in this experiment anyway, I tried not to let the food or what I've eaten or the amount of food eaten change. I tried to keep in mind the time itself to take more of a change body wise, but food wise I ate the same food. And also I tried to eat the same quantities.

(6) did it affect your sleep in any way?

TRF124: I feel like it… it didn't change the pattern, I didn't go to sleep much later or earlier than usual but no, I was going to sleep probably at the same time, but I was sleeping better. I was falling asleep much quicker and not waking up at night, sometimes i used to wake up but this time I slept through. I think in some ways when I was eating before I go to bed it affected me badly. Then I would wake up with a stomach ache, then this time that wasn't happening.

TRF064: I think no because I slept around the same times.

(1) On how many days of the week were you able to comply with the 1.5-hour delay to the timing of first energy intake in the morning?

TRF028: "Breakfast was hard to delay, sometimes I have a lecture at 9am so I cant delay that. But dinner is quite easy"

Commented [JPW(-SoB&18]: Adherence:
Breakfast hard to delay - lectures
Dinner was easier

TRF038: Probably about 5 days of the week, they were normally Monday Tuesday, Weds and Thursday and Sunday.

Any reason why that was the case?

TRF038: Well, Friday night, my husband finishes work at 6 so we have the evening together, and same on a Saturday. So my husband is out in the week, so Friday and Saturday night we tended to have a couple of beers together and eat our meal together.

Commented [JPW(-SoB&19]: Adherence: Weekends unable to adhere - social life, family life eating with partner

TRF097: With breakfast every day, yeah. Because even if I am at work I can easily find a 10 minute break to have my breakfast.

Commented [JPW(-SoB&20]: Adherence: Breakfast everyday

TRF008: I think, apart from when I was abroad, every morning.

What enabled you to achieve that?

TRF008: I think just because I'm not hungry in the mornings. And maybe it's because I start work quite quickly after waking and I'm quite busy.

Commented [JPW(-SoB&21]: Adherence: Morning - Everyday - apart from when abroad Usually not hungry in the mornings

TRF124: every day.

What enabled you to achieve this?

TRF124: I could work from home so before I would just wake up and get coffee with honey, or if I were hungry id eat something, just wake up but normally it was just coffee and honey. And then I just had to delay that by 1.5 h so it wasn't that hard. Also because I work from home I can manage my schedule well.

Would it change if you were in an office?

TRF124: it might have done. Yeah, psychologically I might have been like I need to get my energy before going to work.

Commented [JPW(-SoB&22]: Adherence: Morning - everyday - working from home could manage schedule well

TRF064: every day. Oh, well, sometimes I would eat a bit later. But it was still delayed. So, what I started doing was bringing breakfast into uni so when 11am or later hits I would have breakfast there. During the weeks it is much easier because I start my day later anyway during the weekend, so yeah eating later was not much of a problem. So yeah I started packing coffee and breakfast in the morning and taking it in in the morning.

Commented [JPW(-SoB&23]: Adherence: Morning - everyday - took breakfast into work with them

(2) On how many days of the week were you able to comply with the 1.5-hour advance to the timing of last energy intake in the evening?

TRF028: "I finished my dinner before 6pm and went with no drink before I go to bed.

TRF038: 5-days, oh yeah, I did it five days both ends. I did find eating at 6 quite easy because I had the children and it fitted in quite well there.

TRF097: Considering 7 days, maybe 4 or 5 days.

What were the days you were unable to follow it?

TRF097: It depends on the day, but some days I have to stay in the lab late, so it wasn't always the same. It was mainly work that meant I couldn't follow it or waiting for the train to get home.

TRF008: Again, I think that when I was on holiday for 4 days, no. but then the rest of the time except for maybe 1 or 2 evenings throughout the study, I did it. I reckon at least 80% of the time, if not more in the evenings over the 7 weeks.

TRF124: I would say every day, the exception was when I went to Italy that was a bit tougher to do, because with family event and family dinner there were a couple of evenings I would eat slightly later than I would do. So my window was between 9.30am and 7.30pm and so I was eating at 8-8.30 and it was that reason, family gatherings.

What influenced the difference between being at home and being away?

TRF124: I could stick to a routine at home, like I could manage. My dinners etch when I was there it was tougher to do because of the influence of the family and friends, you know if they say lets meet up at 8 because the working hours in Italy, they have the siesta then they work until 7.30 so we go and eat something after.

TRF064: I think probably, I tried to do it as much as I can, probably around 5-6 times a week. Sometimes id be like 30 minutes later. But I tried not to go substantially up, where there were social occasions where I think this was maybe once or twice, even when I'm around then id still try and stick to it but maybe drinking because you're also drinking. Throughout that time maybe once a week, I probably went substantially over 3 times over the 5 weeks.

Commented [JPW(-SoB&24]: Adherence:
Evening - would finish dinner at 6pm and no snacks or drinks before bed

Commented [JPW(-SoB&25]: Adherence:
Evening - 5-days - ate at 6pm with children

Commented [JPW(-SoB&26]: Adherence:
Evening - 4-5 days - unable to do it when working late or travelling home late

Commented [JPW(-SoB&27]: Adherence:
Evening - everyday apart from 1-2 occasions.
Harder to adhere when on holiday

Commented [JPW(-SoB&28]: Adherence:
Evening - everyday - exception was holiday with family
Easier to stick to a routine at home

Commented [JPW(-SoB&29]: Adherence:
Evening - 5-6 days a week
Social occasions impacted on 1-2 occasions

(3) Were you able to reduce your eating window by 3-hours overall by choosing your own feeding restriction?

TRF028: "Maybe, 3-days in a week"

TRF038: Yes, I was able to reduce it by 3 hours and it wasn't that hard, but you had to be mindful, and I had to think about the timings, and I had to plan when I was going to eat breakfast lunch and dinner because if my timings were messed up you wouldn't be hungry for dinner, if you ate lunch too late you wouldn't be hungry for dinner. And it was hard when I was on shift at the hospital because things happened, and you cant always go for your break. Sometimes instead of having dinner, id have a chocolate bar on my break and just not eat until the next day.

How did you feel doing that?

TRF038: I didn't feel that bad really, you kind of get used to it don't you I think, going for long periods without food.

TRF097: yeah, considering dinner and breakfast I would say between 4 and 5 a week.

You mentioned that you adjusted your eating window slightly, just in general what did that look like?

TRF097: so sometimes if I thought I was going over the dinner time, I would wait a little bit longer before I had my breakfast and tried to stick with it.

TRF097: I think yes. Usually I get up very early and I had breakfast without feeling like I'm hungry – but now delaying my breakfast I was like I really need food, my stomach was craving for food. I mentioned that I had a few problems with digestion, and I feel it helped a bit with that.

How did it help with your digestion?

TRF097: I think like having dinner a bit earlier than usual helped. I had more time between finishing my dinner and going to bed.

TRF008: yeah, mmm. So at one point I was doing longer in the mornings, 2 h in the morning and 1 in the evening but I mostly tried to stick to the 1.5 h each side. I think a little bit you get guilted into it with the glucose monitors, but that was a side effect of it. I often quite struggle when I make rules for myself but I'm a lot better at sticking to them when someone makes the rules for me. And so I think compared to if I was just doing it for fun, I think that helped to stick to it better. It was definitely only on the odd

Commented [JPW(-SoB&30]: Adherence:
3-h overall - 3 days a week

Commented [JPW(-SoB&31]: Adherence:
3-h overall - achievable but had to be mindful
Required planning - think about when to eat
Hard to adhere to on shift work at hospital.

Commented [JPW(-SoB&32]: Adherence:
3-h overall - 4-5 days a week
Adjusted FW accordingly by delaying breakfast to maintain 12-h FW

Commented [JPW(-SoB&33]: Positives:
Improved digestions

Negatives:
Feeling hungry in the morning

occasion where I would not be able to stick to it in the evenings. It was normally just after dance when I was really hungry, but I did a load of times go to sleep really hungry and I would tell my friends that I'm really hungry! Usually I'm not hungry directly after exercise but an hour afterwards I get really hungry and so having done a 3 h dance lesson at the end I was like yeah I kind of would like some food now.

I think one positive though is that I was probably better hydrated than I normally am because when I couldn't eat I would drink more water, which is a good thing because I would normally lead my life in a perennial state of dehydration. Especially after class when I was hungry id just drink more water.

Commented [JPW(-SoB&34]: Negatives: Having to go to bed hungry - due to leisure activities and being unable to eat before

Commented [JPW(-SoB&35]: Positives: Better hydrated during fasting to reduce hunger

TRF124: Yes. I managed to stick to it most of the time, sometimes it happened where I would delay even further in the morning because I was in calls at work so sometimes I was eating after 9.30am but let's say I had a call at 9 it would go on until ten then I could eat. Maybe sometimes at night, I'd advance it even further. I found the morning easier.

TRF064: yes.

(1) How many days a week would you genuinely consider following TRF now the study is over?

TRF028: "It quite depends on my daily activity, roughly about 2 or 3 days, I can do 12-hour eating".

Why is that?

TRF028: "Maybe I want to be healthier. For example, Monday I am totally free, no lectures, I can finish my dinner earlier, nobody will invite me to dinner or a society or something. But on Friday I finish my lecture at 6pm and will go out for dinner afterwards with my classmates. Some days I am totally free, and I can finish my dinner earlier.

TRF038: Erm, probably, I still stick to it, I stick to the morning. Actually at the moment I cant. So for example before I used to eat my breakfast at home I just have a black coffee at home and on my way to my placement I have my breakfast in my car, so I delay my breakfast as much as possible. Erm but then I am eating at about 8.30 now, whereas before I was eating at 7am. Most evenings I eat with the children at 6 anyway.

I am trying to 5, and I probably do it successfully for 4. On Wednesday I always swim, 6-7, and go in the sauna. Last night I came home and had a non-alcoholic beer and some crisps, but when I was on the study I would not have done that. I felt a bit peckish, my husband was here, I sat down, I was aware I was doing it.

Commented [JPW(-SoB&36]: Favourability: Sticking to it after study - in the mornings

So there is a difference between a study and reality?

TRF038: Yeah because I felt accountable when I was with you, I was logging my food and I agreed to do something, so I wanted to give it my best shot.

TRF097: I still am having my breakfast later in the morning, so I am still following it. And dinner yeah, it depends on the day, but I would like to follow it at least during the week but maybe not the weekends.

How come you are still following it?

TRF097: I don't know, maybe because I am used to it. Yeah I found my routine, I make my breakfast and take it to work.

Commented [JPW(-SoB&37]: Favourability: Sticking to it after study - in the mornings

TRF008: Just if I was doing it for me? I think I would probably consider it as a 4 or 5 day thing. I suppose with these things it is difficult to know whether you would do this forever or just to lose weight or something like that. Potentially it might just be a short term thing, but id say a weekday thing would probably be fine. For me food is a real social thing and at the weekend I would not want to have to restrict timings when I'm socialising and it's a pleasant time to be with other people. I've just thought of something else that would probably change.. when I am on my own at home it would be easier but when my parents are here it would be really hard because we all eat together as a family in the evening so I would find it harder to eat earlier on my own. But I think realistically 4 days but again not counting holidays.

Commented [JPW(-SoB&38]: Favourability: 4-5 days - weekdays fine, weekends harder due to socialising.

TRF124: six days.

Why six days?

TRF124: because normally there is always one day a week on the weekend where like Saturday or Friday I have something on, like with family or taking m y son somewhere and then I would eat a bit later so that wouldn't be 14 hours. Then rest of the day especially when I am at work I can manage myself very well, I think I would stick to it, but there is one day a week I find I would go over the window.

Commented [JPW(-SoB&39]: Favourability: 6-days - one weekend day off for family days.

TRF064: I would probably follow it over the weekend because it is much easier to fiddle around with your feeding window. But during the weekdays, I've just had my breakfast around 8.30 so I have gone back to my previous routine, I feel like breakfast that's the hardest bit, but I don't really have a problem switching my dinner going up, having a bit earlier. It's just the work when you're in the office quite late or something late that gets pushed back a little bit. I think probably the weekend is most realistic for me.

Commented [JPW(-SoB&40]: Favourability: Over the weekend - more scope to adjust FW
Ok to adjust dinner routine, but not breakfast.

What makes it easier to follow on the weekends?

TRF064: it is because I don't have that start time of going into work, I can start my day anytime. I can have my breakfast at 11am and do heavy duty stuff after that. It's just during the weekday everything starts off earlier, I have to walk in and things like that. The weekend it's much more flexible because I can start my day whenever.

(2) But realistically, how many days a week do you think you could follow TRF on?

TRF097: No more than 4, I would say. For sure I would say Monday because I know I would get home on time, I'd say days between Monday to Friday.

TRF124: I would say, if I'm at home, with the little effort I could do 7 days but generally I could do 5 or 6 days.

TRF064: maybe 2 or 3

(3) By how much time do you think you could realistically reduce your eating window by on a daily basis?

TRF028: I think a 12-h feeding window would suit me, because it depends on my time I go to bed and wake up. Usually I wake up at 7 and go to bed at 11 or 12.

TRF097: maybe two hours.

What would that look like?

TRF097: Just because I said, it's more difficult in the evening. I am fine with the morning but yeah, if I have more time to eat in the evening.

TRF008: I think realistically I would want a feeding window that was at least 10 hours.

What would be the reason for that?

Because that would cover for days where I have dance, or Korean I would be able to finish and have some food directly afterwards having had a coffee in the morning for breakfast and having started eating at lunchtime so midday or 1pm. That would be the most feasible for me.

That sounds quite fair

Commented [JPW(-SoB&41]: Favourability - Realistically:
4 days - typically work week days Mon-Fri

Commented [JPW(-SoB&42]: Favourability - Realistically:
5-6 days during the week

Commented [JPW(-SoB&43]: Favourability - Realistically:
2-3 days - typically the weekend

Commented [JPW(-SoB&44]: Favourability - Eating Window
12-h

Commented [JPW(-SoB&45]: Favourability - Eating window
2-h reduction

Commented [JPW(-SoB&46]: Favourability - Eating window
10-h - to cover dance, Korean and enable food after these.

TRF008: As I say, when I have dance my class starts at 7 so realistically I couldn't eat beyond 5pm without feeling sick and so that's quite a short window otherwise.

TRF124: I don't know I should try! 3 hours was good. I don't know maybe I could even do 15 or 16 during the week, I don't know though, I should try. I think 3 is good, less would be even easier but I don't know if you would see any benefits. And more, potentially I could do that a further half hour in the morning and night.

Commented [JPW(-SoB&47]: Favourability - Eating Window
3-h was feasible
Would try 15-16h during the week but 3 is good

TRF064: I feel like I can reduce it. I think in the beginning I used to have my dinner really late but now I'm trying to put it forward because at that time I was going home really late anyway so now I'm trying to push it forward by 1-2 hours, I can reduce it by, dinner wise.

Commented [JPW(-SoB&48]: Favourability - Eating Window
Bring forward by 1-2 h in the evening

(1) What would be the main incentives for you to follow TRF?

TRF028: "I think the main reason is my sleep condition, as it improved when I followed the TRF. When the days I cannot follow TRF I wake up at 2 am or 3am, but when I follow the TRF I did not wake up during midnight, I just went to bed, closed my eyes, at 11 I go to bed and woke up at 8am

Commented [JPW(-SoB&49]: Incentives:
Improved sleep - slept through the night during TRF days - on non-TRF days would wake up in the night

TRF038: Erm, well I did do a bit of reading and I was interest in the way the body responds, the blood sugar and stuff. So I exercise early in the mornings, I don't know, obviously I'm in my 40's so I'm conscious of the menopause and unwanted weight gain, so I do everything I can to stop that so I like the TRF combined with exercising on an empty stomach, combine with just being really mindful about my diet.

Commented [JPW(-SoB&50]: Incentives:
Improving blood glucose control
Reduce unwanted weight gain

TRF097: If they mentioned it would be helpful for my health in general, or if they said it would help me to lose some weight.

Commented [JPW(-SoB&51]: Incentives:
Help with weight loss

TRF008: I suppose if there were proven health benefits, if there was anything that improved with having a restriction like gut health or something. I don't know it's quite tricky because I font usually look for ways in which I can change my diet normally.

Commented [JPW(-SoB&52]: Incentives:
Gut health
General health benefits

TRF124: so wellbeing, the way I feel, also losing weight. Like for instance When I was doing the TRF I lost some belly fat I think, which happened once I started doing it again. Like for health reasons, you measured my glucose and I'd do it for that.

Commented [JPW(-SoB&53]: Incentives:
Overall wellbeing
Weight loss - losing belly fat
Improved glucose control

TRF064: I think when I did it for the study, the incentive was for the study, like what would happen. But personally, nothing much. I think especially during the summer it's just nicer to have dinner a bit earlier, you know because the sun is still out and you can enjoy it outside but other wise nothing much.

Commented [JPW(-SoB&54]: Incentives:
Eating earlier means getting to enjoy more of the warm weather during summer.

(2) What are the main barriers for you to follow TRF?

TRF028: "my friends and my room mates and social media.

Commented [JPW(-SoB&55]: Barriers to TRF:
Social media - food advertisements
Living arrangements - room mates cooking

How does SM affect your eating times?

TRF028: I usually browse Tik tok or Instagram when I go to bed at night, and there are some videos with food on them. And I feel hungry, so I go to the kitchen and get something to eat. When I see advertisement for burger or pizza it makes you really hungry and your desire, you want something to eat.

Has that changed the way you use SM?

TRF028: I read books instead of social media! Much better!

TRF097: I think maybe my lifestyle, my work, my day and how I plan my day.

Commented [JPW(-SoB&56]: Barriers to TRF:
Work
Lifestyle
Day-to-day planning

TRF008: I mean, I think with anything, when it gets complicated it makes it harder to follow. So when there are lots and lots of rules, obviously if its just time there wont be but its easier for me when its clear you eat between x and y rather than… I like flexibility but with rules. When there are too many it makes it challenging. i think the biggest thing is when it starts to encroach on your social life which mostly for me are the times to meet friends and chat and share experiences, and you usually meet at an eating establishment.

Commented [JPW(-SoB&57]: Barriers to TRF:
Complicated protocol - too many rules
Social life

TRF124: I think I'd be able to follow TRF most of the time, so barriers as I mentioned earlier might be social events that you have to go to or work events that would make it a bit more difficult but most of the time it shouldn't be a problem for me. It's more like the motivation or you lose the routine.

Commented [JPW(-SoB&58]: Barriers to TRF:
Social events
Lack of motivation
Loss of routine
Work trips
Family visiting

After the study you said you carried on with the routine for a bit then you dropped off, why did you drop off?

TRF124: I went away with work for a week and that totally ruined my routine. Then I came back and my sister came over and stayed with me for a while and that was her influence and you know cooking, it was still a social motivation. But now I cant wait to go back to it. I definitely feel much better when I don't eat later at night so my last meal is at 7pm or 6.30 and then that's it.

TRF038: Well work commitments, some placements I have to work into the evening so that makes it more challenging. But not impossible. And also lack of time because also I think you have to prep for it, I sort of take my breakfast with me, my lunch with me and I have my dinner waiting for me when I get home. Well the occasional social event, but I don't really go out that much but when you do go out, it's very hard because you usually go out for 7.30, but that's quite rare for me. I might go out once a month, its usually at the weekend.

Commented [JPW(-SoB&59]: Barriers to TRF:
Work commitments - working late into the evening on placement
Lack of time - due to prep for it
Social events

TRF064: apart from work and social gatherings, that was mainly it. But if I am more flexible with work time then maybe I can change my feeding windows as well but it's mainly work really.

Commented [JPW(-SoB&60]: Barriers to TRF:
Work
Social events

(3) Do you have any other comments to offer?

TRF028: Maybe next time, you can investigate people's mood change, not me but other people. As I know In China, research shows less people get anger or depression when they do TRF.

TRF028: "I found that I ate more food containing protein when I ate rice or pasta I would feel hungry. But if I ate eggs before I go to bed I would feel less hungry. I was more conscious at dinner to eat more protein, to feel less hungry.

Commented [JPW(-SoB&61]: Impact of TRF:
Ate more protein at dinner to feel less hungry

TRF038: I do not know who the study, what the outcome would be, but I think to do TRF you have to be quite disciplined, and I don't think the generally population would be up for it. Well they would be up for it, but I don't know whether they would carry it out. I am in a close group of 8 girlfriends and we're all about the same age, and when I told them what I was doing and we went away one weekend and I had the CGM on my arm, they were like oh my god how could you do that, why would you do that? I am probably the healthiest, fittest, slimmest out of all of them, and they would gain more from it but thy thought it was too restrictive for them.

Is that how you feel?

TRF038: Yeah I sort of feel if I weren't so invested in my health and keeping fit for my children, I don't think I'd have the motivation to do it.

Commented [JPW(-SoB&62]: Negatives of TRF:
Requires discipline
Need to be motivated to change

TRF097: I think if I were to do it again, I would do the full 3 hours in the morning and keep the evening to be able to eat just because of my own timetable. i think for my schedule personally that would work a bit better and that's where it has to be flexible to suit different lifestyles. I think also in the exit survey it asked how many days would you do it, and I think that would be the other thing that would help knowing that you have a couple of days where you knew you had something going on and you knew that you didn't have to be so strict then I think it would be more easy to follow. I find that any of these changes in diet only work if you are happy to do them and you are able to fit them to your lifestyle and I think you do have to be a bit flexible and you might have a couple of weeks when you are away or going to weddings or parties and you cant restrict your intake. So yeah, having some kind of flexibility is necessary for any kind of long term dietary modification.

Commented [JPW(-SoB&63]: Favourability of TRF: Delay for 3-h in the morning and have flexibility in the evening.
Having free-days would make it easier to follow

TRF008: honestly it was easier than I thought it was going to be and it showed me that I can be more in control of when I am going to eat and when I'm not. But it also reminded me how much I dislike recording everything that I eat it just doesn't make me feel very good. I know its not a big deal but to me its very personal and I'm like oh yes this is why I've never done this before. I think it is doable and it seems more feasible that other TRF things I've heard my friends do. So yeah I think overall I think it was an interesting experience and I think nothing about it was bad.

Do you know what some of your friends were following?

TRF008: oh yeah, some of them were doing the 16:8 one.

TRF124: Only that I found it really good, I would love to see from you study if there was a real benefit for me or a placebo effect. One thing I wanted to mention, sometimes, I would eat more during the day, probably just because…at the beginning I just thought I only have this time to eat, and I cannot eat later so I'll eat more now. But then I stopped doing that.

TRF064: Nothing really. I feel like the only thing I struggled on was the morning, but otherwise it was fine. I feel it also motivated me to keep me on track, and I guess having you in the office! I think it was just the breakfast I struggled on the most. But otherwise it was easily compliable, and it was not too hard basically. Otherwise if I knew it was going to be very difficult on me, very extreme, then I probably would not have entered the study anyway.

Control group

(1) How do you think you would find following the TRF intervention?

TRF024: "I think I would find it, challenging at times, I think it would definitely be doable, but I definitely think it requires some planning and prior thought into how you are going to plan your day around eating. And it terms of socialising I think I would find that difficult. A big part of uni is about going out, meeting people, having a few drinks, which doesn't work within the study, but no, if I had enough willpower I think I could definitely do it.

Commented [JPW(-SoB&64]: Potential adherence: Challenging at times but doable

Commented [JPW(-SoB&65]: Negatives of TRF: Requires planning when you are going to eat.
Social events

TRF015: I think after a period of time I would be able to adapt to it well. I think the bit I would struggle more with from a personal standpoint, is the shortening in the evening. Because I am a terrible snacker, that would be the area that I find more difficult. In the morning there would be no issue whatsoever I don't think. The evening aspect would be more of a habitual change then actually an issue to undertake. It would just be training myself to do so.

Commented [JPW(-SoB&66]: Adherence: Difficult to advance in the evening - habitual snacking
Morning easier

So there is no other reason why you snack more so in the evening?

TRF015: "it's purely just habit, I think a lot of it comes because we usually are eating a meal with the kids, so we are eating earlier than we might do normally. As a result I'm then peckish later and it's easier to grab something, I think it's just the self-control aspect. Eating that main meal we probably do already, but its about cutting out those habits more than anything else.

TRF123: I think I would have beem fine, my only struggle is like, I can delay my breakfast as long as I want, I can make dinner earlier or later I just get a bit bored in the evenings and that's why I snack, its not like im eating actual meals I'm just eating out of habit. I think I would struggle because I struggle to sleep and I have this thing in my head that I cant sleep if im hungry and end up eating really late. I think that would be the only thing that would stop me, but I think that's just a mental thing. If I actually put my mind to it I could follow it quite easily.

Commented [JPW(-SoB&67]: Adherence: Morning delay easy
Evening advance difficult - due to habitual snacking

TRF126: I think the fact that I was in the control group was a blessing in disguise because it would have been quite difficult for me to follow the evening restriction more so than the morning. The morning would have been fine but if it was to be for 7 days a week for the full duration of the intervention I think I would have struggled on a few days purely because I think socially there are commitments especially over such long period of time that I wouldn't have been able to get out of and I think it would have been difficult to stop at that time. But any other day in my normal routine, and let's say I have dinner normally at 8.30pm, I potentially could have stopped on the normal days at 7pm, that would have been doable. But also saying that I was working from home for most of it whereas being in the office that might have been problematic because the commute would have delayed getting

Commented [JPW(-SoB&68]: Adherence: Evening advance difficult
Morning delay easy

Commented [JPW(-SoB&69]: Barriers to TRF: Social occasions
Working in the office and commuting

home and then being able to eat. I think overall I would have found it quite difficult to follow if it was 1.5 h at each end of the day.

(2) What do you think the negatives of following TRF would be?

TRF024: "I think that to have an evening meal quite early will almost disrupt the schedule of your day, I would find. I think just being quite constrictive I would find that difficult. I don't think I'd find the morning very difficult; I think that would be doable.

Commented [JPW(-SoB&70]: Evening meal difficult

Commented [JPW(-SoB&71]: Morning easy

TRF015: "I think that niggling hunger in the evenings, and you know, if youre not going to bed until later in the evening then it just elongates that period and that could be where temptation comes in. You tend not to eat healthily I would say, you're not maintaining that balanced diet necessarily when you're snacking, regardless of what you are snacking on, I know I certainly don't. For me it's usually biscuits and cheese or something like that, which is probably the worst thing I could be eating at that time of night. That would be the hardest thing to get used to.

Commented [JPW(-SoB&72]: Negatives of TRF: Increased hunger in the evening - leads to temptation and unhealthy snacking.

TRF123: like I said, for me, I think from when I was younger when I couldn't sleep and my mum would say have some food, I've just been conditioned into thinking if I'm remotely hungry then I won't go to sleep, and sleep is difficult for me anyway so I really struggle not eating after dinner, so I would have a struggle initially adjusting to the fact that I can sleep if I feel hungry.

Commented [JPW(-SoB&73]: Negatives of TRF: Preconception of not being able to sleep when hungry, and TRF exacerbating this feeling

TRF126: Well, it's in the title really, you are restricting yourself, not just on the time but on what you would be doing in the evenings I suppose or just your general routine and I suppose that is where there is that element of weight loss for some people because you are stopping yourself from doing those things that generally happen in the evenings. It's the preferred time to see people as opposed to lunch time, so I think that would be the main thing.

Commented [JPW(-SoB&74]: Negatives of TRF:
Restricting the times you can eat
Impacting on social events
Disruption to routine

(3) What do you think the positives of following TRF would be?

TRF024: Just that it would be easy to follow, like there's no complex idea behind it which I think would appeal to people. Yeah, that would appeal to me, but I just don't need to do that.

Commented [JPW(-SoB&75]: Positives of TRF:
Easy to follow

TRF015: "I think given time, obviously, the theory behind it and being part of the trial, it seems logically, a healthier way to eat, to give your body time to digest. To start that digestive process before you go to sleep, so I would imagine when you think about the absorption of fat I can see if you have a balanced social activity and fitness regime I can see that being really healthy.

TRF015: I think one of the real benefits of it, is by compressing that meal window slightly through the day, you're not going to have that time where you feel suddenly really hungry. And I think that would offset that concern I had earlier where I was saying that hunger in the evening I would hope to think that hunger period is quelled.

Commented [JPW(-SoB&76]: Incentives of TRF:
Healthier way to eat - by giving the body more time to digest.

TRF123: I think probably, it would be weight loss. It's something I've looked into a lot and there's mental health benefits, right? I think I'd probably eat less crap so I'd be healthier.

Why do you think you would eat less bad foods?

TRF123: because like I said, through the day I eat relatively healthily and then after I have dinner I just eat like, but then because I'm not really hungry I just eat a sandwich or some crisps just things like that and I think if I focused on the 12 hours, that's when I eat the junk food, I wouldn't be eating the junk food anymore.

Commented [JPW(-SoB&77]: Incentives of TRF:
Weight loss
Mental health benefits
Reducing the amount of junk food consumed in the evening

(4) Do you think it would affect your day to day living in any way?

TRF024: "I think that I would get a bit stressed about my evening meal, because I would have to be eating at about 5.30 or so. I wouldn't finish uni work until 5 so it gives me half hour to make something and eat something and I think that only gives me a small window of time really.

TRF015:" I think it would just be about being more disciplined, in how I eat. I think I'm very inconsistent with the times that I eat after breakfast, so we have a pretty set routine getting up kids and if I'm going to work then breakfast is at a consistent time but then I'm really bad at making sure I eat lunch at a consistent time and I might be earlier, as a brunch type thing, then a really long period before I eat in the evening. Or more often than not I would work through lunch when my body would tell me it would be and then have a later lunch and again probably push that final intake later because I'm not hungry then. So you know, instilling that self-discipline and actually having that more fixed mealtimes would be one of the difficulties I have in maintaining it.

So there needs to be an element of flexibility but at the same time, it needs some organisation?

TRF015: "yeah absolutely, when you have undertaken it, and being part of the trial and considered how it would fit in with my lifestyle, that is the thing that would be the most problematic is the restriction in flexibility. But actually there are other things I can flex around that, what do I prioritise? It would just be a shift in prioritisation to make sure that I can maintain that routine around meetings rather than flexing when I eat around meetings. When you say it out loud it seems so backwards, when you think about a healthy lifestyle, but I think we're all quite guilty of that, you know how we balance work-life pressure and stuff.

TRF123: no not particularly, I mean I work from home every day so it wouldn't be a struggle for me to adjust my eating habits.

Do you think that would be different if you were in the office a bit more?

TRF123: erm, only that I know that when I've had jobs and gone to work I'm much hungrier. But yeah, no, I don't think it would be that difficult.

TRF126: the positives, well I suppose once I see the result and I know there are positives, there wasn't really anything I felt that was different. I think the positive would come in the results from the data. I suppose you may feel less lethargic when you don't eat later into the evening, I wasn't going to bed feeling like I was still digesting, although I know I was in the control group, I know that from in the past when I have had time to digest before I go to bed then I sleep much better.

(4) Do you think it would affect your food choices in any way?

TRF024: "Erm, well it probably would because I think I would want to have something that I could cook faster. But I also think I don't know whether or not you'd have the same level of time to build up the same degree of hunger so you may eat less

TRF015: Quite possibly, I think portion control would probably go out of the window. I think by that point you'd get to that point where I say famished in the loosest sense of the word, but if I'm delaying first intake at that point then my first energy intake is going to be a lot bigger than it would normally. I'm also more like to try and grab quick food so stuff that's pre prepared or something like that which is inevitably less healthy, higher in saturated fats. So I think that's the kind of inference of that, just the ease rather than try to make something healthier.

TRF123: maybe, I'd make more conscious decisions about getting the good calories in during the day so that I wouldn't… I tend to have quite small meals then I snack in the evening so then if I was really trying to stick to it I'd be thinking about sustenance from the meals in the day so I wouldn't feel hungry later.

TRF126: I think perhaps it would make me, well, if I was doing it long term it would make me more selective about when I was choosing to socialise in the evenings and seeing people earlier on so that I could make it feasible. I also think perhaps I would choose to eat foods that are slow release energy that will keep me fuller for longer instead of eating junk food because I don't want to be starving again before I go to bed and that period of time could be quite long. I mean it would stop me choosing to snack on things in the evening and I would eat them earlier on in the day.

What sort of foods are you referring to when you mention snacks?

TRF126: Erm the junky stuff like the chocolate and the ice cream because that's one of my weaknesses in the evening. So I would either have them on in the day which is unlikely, because it's just not part of my habits but choose to eat something a bit more substantial and nutritious because it would keep me a bit more fuller for longer and I know I can't just grab that.

(6) Do you think it would affect your sleep in anyway?

TRF024: " I think, I cant sleep if I'm full so I think it would help, but I also think if I was incredibly hungry I would wake up". But I'm sure I wouldn't be absolutely starving.

TRF015: "I don't think so. When I think back to pre-children, I think if I ate too late you would always feel veery bloated, very heavy when you went to sleep, and getting to sleep could be more difficult potentially sometimes. I know when I eat earlier I almost feel ready to sleep, it's like your body is not preoccupied doing anything else, the flipside is that if I eat a really heavy meal you want to go to sleep but I'm not sure its quality sleep, it's almost like a food coma. I think there is the potential there, and if I was undertaking TRF, I'd be doing it looking at it from a health perspective for me, and with that comes more conscious choice about what I am eating and I think hand in hand that would come with a better sleep pattern and a better circadian rhythm.

TRF123: I think initially I would probably struggle to sleep, but like I said, I'm really bad at sleeping anyway. I probably am not but I just have this mental block in my head.

(1) On how many days of the week do you think you would be able to comply with the 1.5-hour delay to the timing of first energy intake in the morning?

TRF024: "Probably 5 or 6. The only thing that would stop me would be if I weren't very organised and I had something on in the morning, and if it was 3h long I would rather eat before, than have to sit through and be hungry. I think since doing the study I have been a bit more mindful of my hunger levels in the morning and I realise that it does take a while before I even feel the inclination to have breakfast.

Commented [JPW(-SoB&78]: Adherence:
Morning - 5-6 days
Would be difficult if not organised or had events on in the morning

TRF015: "I would say easily the 5 working days if that makes sense. I would adapt, probably take it to work to eat. But at the weekends at home it becomes a little bit more difficult simply because I've not got that separation from the kids, so you know, maintaining a routine for them is often crucial to then follow through with things. If I'm suddenly not eating breakfast with them then it seems a bit odd to them. So trying to fit it around family routines around weekends might be more difficult whereas if I'm in the office I can just take it with me.

Commented [JPW(-SoB&79]: Adherence:
Morning - 5 working days

TRF123: I think I could do it every day. Breakfast is a relatively new thing for me anyway, so I always used to skip it just because I prefer to sleep in the mornings. Having to get to work, I've only been having breakfast since I've been doing my PhD, and also trying to be healthier because I've been trying to lose some weight for a while now and I think eating breakfast is better. But I think I can skip it because I can get through until lunch time as I

Commented [JPW(-SoB&80]: Adherence:
Morning - every day

think the struggle comes after that. I think maybe it just delays me wanting to have food so if I dont have any food so the only trouble with me is like concentration and stuff.

TRF126: at a push, I would say on working days I could postpone it, because those mornings I wouldn't generally sit down and have my coffee until I start work so in the week I could potentially do that. But on the weekend it might be more difficult because I still walk up early and I don't have too many things to keep me busy.

Commented [JPW(-SoB&81]: Adherence: Morning - working days only

On how many days of the week do you think you would be able to comply with the 1.5-hour advance to the timing of last energy intake in the evening?

TRF024: "Maybe 2 or 3 I would say. Just because of plans, social plans or just, I eat lunch at 2pm so firstly, I would probably have something on which would make it difficult to make time in my day to make a meal and also I wouldn't be hungry. I mean I might have a snack around 5pm or 6 but I wouldn't feel ready for a meal.

Commented [JPW(-SoB&82]: Adherence: Evening - 2-3 days - due to social plans, late lunch = lack of hunger

TRF015: "again, I think that might be quite difficult for us. With the routine the children have, but I think because we are eating earlier, I would like to think I fall within the optimum by delaying the breakfast.

Commented [JPW(-SoB&83]: Adherence: Evening - difficult - due to children routine eating at 6pm anyway

TRF123: I think probably Monday to Friday I could then weekends it would be more difficult just because of going out and stuff, socialising.

TRF126: Yes, that goes back to what I said earlier and if I am working in the office potentially that could be slightly problematic because I wouldn't get home in time. However, those are also evenings where I try to meet people after work in town so potentially I would have an earlier dinner and I would work around that. Alternatively, on the other days I would generally go out once or twice in the evening those are the days I would find difficult. I would say overall, I could do it for 4-5 days and have 2 days off.

(2) Do you think you would be able to reduce your eating window by 3-hours overall if you were able to choose your own feeding restriction?

TRF024: " I think, yep, that would be doable, but I don't think it would be something I will be implementing into my life though, but I think it would be definitely doable.

TRF015: "It would be a shift, morning to later, aligned to that. It's not to say that we can't shift in the evening but to do such a dramatic change would be quite disruptive to our routine and that might create unnecessary tension that I want to avoid.

How would you achieve this?

TRF024: "Could I push back, not eat breakfast for 2 hours then eat dinner an hour earlier. I think that would be how I would do it".

Commented [JPW(-SoB&84]: Adherence: Evening - Mon-Fri and free-days on weekends

Commented [JPW(-SoB&85]: Adherence: Evenings - weekdays on - weekends off

Commented [JPW(-SoB&86]: Adherence: 3-h overall - delay breakfast and eat later

Commented [JPW(-SoB&87]: Adherence: 3-h overall - delay breakfast by 2-h, advance dinner by 1-h

TRF123: I think I would be more likely to because I could just add it on, so I'd give myself more hours in the evening and delay it in the morning.

Commented [JPW(-SoB&88]: Adherence:
3-h overall - delay morning and eat more in the evening.

TRF126: Yes, and I think to fit in with my routine, potentially I would do 2.5 hours in the morning and then a half hour or an hour in the evening. So I think in the week, at the moment I have my coffee around 9.30 so I could push that to 11am and have a late morning coffee because that's really my first calorie intake. and then that would give me a little bit more leeway in the evening. So if I eat at the moment at 7.30 / 8 I could reduce that by half hour or an hour at the most. To eat maybe at 6.30 or 7pm. But then if I was to meet people it would be difficult.

Commented [JPW(-SoB&89]: Adherence:
3-h overall - delay morning by 2.5h and advance evening by .5h

(1) How many days a week would you genuinely consider following TRF?

TRF024: "I wouldn't really, I think when it comes to eating I don't want to be restricted and I'd rather just eat when I'm hungry. And if I'm not hungry, then I'll just wait until I am rather than having hard and fast rules or guidelines that I don't want to follow. I think for me its about being mindful of how I'm feeling and if I'm hungry and when that is.

Commented [JPW(-SoB&90]: Favourability:
Wouldn't follow it

TRF015: I think with certainty and confidence I can say 5 out of the 7 days would be achievable. I like to think we could do more but you know, I think the weekends would be when it would tail off, with activities and socialising and that kind of thing. I would

Commented [JPW(-SoB&91]: Favourability:
5 days - weekends are free days

imagine once you develop the routine it would creep into the weekends, almost like a circadian rhythm for your body, I imagine you'd start to see a slight restriction in how you are feeding on the weekends without intending to because your body would adapt to that feeding cycle.

TRF123: I'd consider doing it every day, but I'm just thinking about days when I go out drinking, like right it's 8pm and now, you know. Days when I wasn't doing that, normal days, then yeah, I could.

Commented [JPW(-SoB&92]: Favourability: Everyday apart from on days when going out drinking

TRF126: if I really pushed myself I would say 4-5 days. But then as a person I don't like specifically eating routines where it changes over the week. I don't like the 5:2 diet where you have days on and days off I would just rather be consistent every day, so I don't know if this would be well suited for me as an ongoing thing or something I would pursue.

Commented [JPW(-SoB&93]: Favourability: 4-5 days

Is that because of your varied lifestyle?

Well, I just think day to day I wouldn't want to discipline myself where it's based on time. I would want to look at what I'm eating perhaps and change that rather than basing on the time of the day or how much I can eat within a window.

(2) But realistically, how many days a week do you think you could follow TRF on?

TRF123: I would say 5 again, it's basically Fridays and Saturdays would be a struggle but Sunday to Thursday would be fine. I've basically stopped having snacks and staff after doing your study, I was talking to my supervisor as well and for some reason I was like I

wish I was in the experimental group and she just said just do it! So I've been doing it for just a couple of weeks. I've actually been sleeping a lot better, going to sleep more easily.

TRF126: But if I were told to do it, I would say realistically I would do 4 to 5 days.

(3) By how much time do you think you could realistically reduce your eating window by on a daily basis?

TRF024: "2 hours would definitely be doable, I think.

TRF015: "I think easily shifting it an hour each side would be achievable, and I know half an hour in the grand scheme of things sounds very silly, but just doing that it goes from eating 5 for the kids but feed them closer to 4pm. That would be quite achievable without causing massive disruption whereas if you move it an hour and a half forward you're barely home from school.

Did you say that you eat with your children? What time do you tend to eat dinner?

TRF015: "Yes, usually we eat about 5pm. So we eat quite early so when I think of that restrictive window to bring it forward I'm eating at 3.30pm! but that also precipitates my bad habits with food because we are eating earlier but actually we've made the decision for us as a family unit, but we've noted the kids eat much more effectively when we eat with them than when we eat separately.

Commented [JPW(-SoB&94]: Favourability: Eating window
2-h reduction

Commented [JPW(-SoB&95]: Favourability: Eating window
2-h each side

TRF123: probably not loads, a long time ago I tried doing a 6 or 7 window and it lasted for like 3 days because it just wasn't enough.

Why did you find that difficult?

TRF123: I just felt so tired all the time, and concentration was really bad. I think from what I understand, you have to just push through that and then feel the benefits afterwards, but trying to write a PhD and stuff when you cant concentrate is really difficult.

TRF126: I find it difficult to reduce my evening window anyway, although I know it's better to eat dinner earlier and I do try with my partner to eat as early as possible, but it is quite difficult to do that in the evenings. I think the earliest we have managed to eat is 6.30pm and that is very rare. So realistically, nothing from the evening side but from the morning, and I don't know if this is a good thing because I am always told off for not eating anything in the morning, I could maybe have my first coffee of the morning at 11ish instead. That's realistic, but by then I do definitely need something because I couldn't wait until lunch. I could potentially reduce by 2-3 hours depending on whether it's the morning and the weekend.

Commented [JPW(-SoB&96]: Favourability: Eating window
2-3h depending on whether it's the morning or weekend

(1) What would be the main incentives for you to follow TRF?

TRF024: "I know that for a lot of people weight loss would be the main reason but for me that's not something I'm particularly desiring. I think definitely better sleep because I know pushing my meal forward a bit might help.

Commented [JPW(-SoB&97]: Incentives of TRF:
Better sleep
Weight loss

TRF015: "With me with a family unit, everything has to fit around that because they become more of a priority than yourself. So whatever is effective for them and I can fit around that then that's perfect. And because we are eating earlier, that side of the restriction, is probably already

undertaken. The other side is an adaptation I can make without an impact on them on weekends kind of thing. Because if I tried to stop them eating before 8am then they'd lynch me!

I think from a weight maintenance aspect that is something I would consider, and certainly as a diet aspect as I am getting older the health factors and risk factors are something that I probably should be more aware of and I am at the moment, and I think with the whole psychol-social aspect of being aware of eating I think that hand in hand would come an awareness of what you are eating. Particularly at first anyway making sure you don't eat too late and don't eat too early I'd be more aware of what I am eating just as an effect of the TRF. If you are going to the effort of controlling when you eat it seems to me that you would be aware of what you are eating because I don't think it would come down to convenience at that point in time.

TRF123: I think probably mental health benefits, from what I've read about, maybe also some slight weight loss but I don't really need to lose weight but I can notice myself putting on weight when I always snack. Mostly for me the mental health benefits, I'm always on a quest to improve my mental health.

What sort of MH benefits are you thinking of?

TRF123: I think mental clarify, reduction in anxiety and sleep is always a thing. I don't know if what I just read is true but I heard it makes you feel more alert.

TRF126: I suppose it makes you more conscious of what you are consuming and it definitely breaks bad habits. I think that would be a good incentive because people will often find it difficult to remove things from their diet and I've been one of those people who have been told you cant eat this or that, but if you're just told your time is up and you cant eat for the

Commented [JPW(-SoB&98]: Incentives of TRF:
Weight maintenance
Family time increased
Increasing awareness of diet

Commented [JPW(-SoB&99]: Incentives of TRF:
Mental health benefits
Weight loss
Reduced anxiety
Sleep quality
Alertness during the daytime

Commented [JPW(-SoB&100]: Incentives of TRF:
Makes you conscious of what you are eating
Helps to reduce bad eating habits

rest of the day it's a no brainer. It doesn't matter what you can and cant eat you just have to stop thinking about it and maybe that's a bit easier to process. I think that would naturally have a knock on effect about being more selective about healthier eating because itll keep you going for longer. And maybe eat more regularly over the period when you can eat because you appreciate it more, rather than thinking I'll eat at some point so it doesn't matter, so it may encourage me to eat more during the day.

(2) What are the main barriers for you to follow TRF?

TRF024: "Time constriction would probably be the main one. I also use a shared kitchen and it's quite small, so I have to plan my meals around when others aren't in the kitchen, just for space. I guess access to cooking or food facilities as well.

Commented [JPW(-SoB&101]: Sharing a kitchen, cooking around when the facilities are free

TRF015: I think honestly, probably work. And you know, keeping the rigidity around that to ensure you have secured time that you can bring that later meal forward if you are staying in the office or anything like that. Likewise, if you have a jam packed day and on 9 or before 9 you are busy or teaching, then that would impact when I could delay that meal to. The inference being that I either eat before I start teaching or I wait until 11am when we break normally to have some food intake. I don't think that's an absolute obstacle but I know that by that point I'm going to be really hungry and then obviously push lunch back, which isn't the end of the world but you end up really restricting your feeding window.

Commented [JPW(-SoB&102]: Work as a main barrier.

TRF123: honestly, because it's like a coping mechanism also, I gave up smoking a few years ago and I am trying to really reduce my alcohol intake so they are the things I had as a

Commented [JPW(-SoB&103]: Food as a coping mechanism

coping mechanism so now if I get overwhelmed or something I immediately want to eat stuff. And my life is really high stress, family life and stuff like that I find myself eating a lot in the evenings also because it's a lot of like stress, so I think a barrier for me would be trying to find something healthier to replace that.

TRF126: it's mainly the evening restriction and it just doesn't fit in with my life. You know if I was a mum with young kids potentially it could be ideal because I'm not going to be doing anything in the evenings. But I work in a very busy city and I see a lot of people, I'm a very sociable person that would be quite problematic. So it would just clash with my social life.

Commented [JPW(-SoB&104]: Evening restriction, social life, doesn't fit in with TRf

(3) Do you have any other comments to offer?

TRF024: "I do like the idea of it, and it doesn't sound like the most rigid of plans, and I know there are some fasting plans where you do 24h fasts and that's obviously not quite as feasible, so I think for someone who's trying to maybe lose weight or to try and maybe have a better eating relationship then it definitely might be more viable than some of the other proposed diets. I think for lots of people losing weight, there is a lot of misinformation out there and a lot of people don't necessarily come across good sound nutritional advice, backed by science and there are so many fad diets. I think it's so important that people, if they are going to undertake something like this that they do understand that there is science behind it and fact behind it. Rather than it being touted as the one solution which I think lots of things are." "I did find it interesting that you said it was mostly females that were interested in this, erm, I think anything to do with weight loss and society, well females, to be slim, is quite a large pressure there. People are toted as an ideal really, but for men, they have a different idea of what their physique should be like so instead of weight loss is more like building muscle."

TRF015: From my healthcare background, I'd be cautious of everybody doing it. If you have health conditions, somebody who's managing diabetes for example, and glycaemic control, I think there's considerations to have about that and how people manage that. I think it is a relative obstacle, if you have someone who wishes to do it for the benefits of it, if it's shown, then actually you manipulate the food you have to maintain that glycaemic control and you manage your insulin intake accordingly. I think I'm relatively active, I'm not as sporty as I was, but when I am playing a sport I do manipulate when I eat so I know I have good energy levels and it is well digested when I play sport, I think again that might influence how people might adhere to it.

TRF126: it's definitely been interesting even though I was in the control group, it was interesting to become aware of what I'm eating but not in the content of what I'm eating but more looking at the time window was very alien to me. It sounds quite easy in theory but in practice it's a bit more challenging, but it might encourage you to eat healthier throughout the day. It does have this knock on effect and if I had been in the TRF group I think I would have seen massive changes, and I think I would have seen the weight loss, I think I would have had the energy because it's the time restriction but then it involves into just trying to eat better and cutting out…so I like a drink and I drink in the evenings generally because I cant drink in the day and I'm working but that would have reduced my alcohol consumption which is of course a huge thing so I can definitely see that it would have changed but realistically I don't think I could have stuck to it for the full 5 weeks but I know it would have made huge changes to my body composition and just to what I was consuming every day. It would definitely be a beneficial exercise. Final comment, I would say it's very different to what I've ever considered before and I think it could be quite refreshing for people to look at if they are struggling to achieve certain goals, this could be the one that works for them.
